# The ApWRKY26/ApERF4-ApMYB2 module regulates anthocyanin accumulation for the seasonal leaf color transition in *Acer palmatum*

**DOI:** 10.1093/hr/uhaf257

**Published:** 2025-09-24

**Authors:** Zhu Chen, Faheem Afzal Shah, Xiaoyu Lu, Lu Zhu, Guo Wei, Xin Meng, Qiuyue Ma, Jie Ren

**Affiliations:** Agriculture Mechanization and Engineering Research Institute, Anhui Academy of Agricultural Sciences, Hefei 230031, China; Agriculture Mechanization and Engineering Research Institute, Anhui Academy of Agricultural Sciences, Hefei 230031, China; Cultural & Creative College, Anhui Finance & Trade Vocational College, Hefei 230601, China; Institute of Leisure Agriculture, Jiangsu Academy of Agricultural Sciences, Nanjing 210014, China; College of Horticulture and Landscape Architecture, Yangzhou University, Yangzhou 225009, China; Agriculture Mechanization and Engineering Research Institute, Anhui Academy of Agricultural Sciences, Hefei 230031, China; Institute of Leisure Agriculture, Jiangsu Academy of Agricultural Sciences, Nanjing 210014, China; Agriculture Mechanization and Engineering Research Institute, Anhui Academy of Agricultural Sciences, Hefei 230031, China

## Abstract

*Acer palmatum* ‘Duocai’ is an excellent ornamental cultivar maintained through asexual propagation. In spring and autumn, it exhibits red leaves, and in summer, it displays green leaves. To investigate the genetic and epigenetic regulation underlying these seasonal pigmentation shifts, we implemented a comprehensive multi-omics approach. Metabolomic profiling identified cyanidin-3-O-glucoside as the predominant biochemical factor governing seasonal leaf color transitions. RNA-seq, ATAC-seq, Hi-C, and WGBS were utilized to examine transcriptomic and chromatin remodeling dynamics. Multi-omics regulatory network analysis identified ApMYB2 as a key transcription factor (TF) affecting anthocyanin accumulation by regulating *ApF3'H2* expression. Functional analyses demonstrated that the TF ApWRKY26 positively modulates *ApMYB2* expression, while ApERF4 exerts an inhibitory effect on its expression. These regulatory interactions were corroborated by seasonal RNA-seq-based correlation analyses. Genetic manipulation experiments, including overexpression and silencing of these genes in *A. palmatum*, provided empirical evidence supporting their functional roles in the anthocyanin biosynthetic pathway. Together, our study elucidates the molecular mechanism by which ApWRKY26 and ApERF4 coordinate the activity of *ApMYB2* to govern seasonal anthocyanin synthesis in *A. palmatum* foliage.

## Introduction

*Acer palmatum*, widely cultivated as an ornamental species throughout East Asia, is prized for its distinctive leaf morphology and striking seasonal color transitions [1, 2]. These chromatic shifts are primarily governed by the dynamics of foliar anthocyanin accumulation. Anthocyanins, as natural pigments, play crucial roles in regulating the coloration of diverse plant organs and enhancing stress resistance. Structural diversity within this pigment class—exceeding 600 variants derived from six core aglycone backbones (cyanidin, delphinidin, pelargonidin, peonidin, petunidin, and malvidin)—is characterized by cyanidin-based derivatives dominating red-pigmented tissues. Anthocyanin biosynthesis relies on phenylalanine as the starting point, with key enzymes driving the process—specifically chalcone synthase (CHS), chalcone isomerase (CHI), flavanone 3-hydroxylase (F3H), flavonoid 3′-hydroxylase (F3′H), dihydroflavonol 4-reductase (DFR), anthocyanidin synthase (ANS), and UDP-glucose transferase (UFGT) [3]. The endoplasmic reticulum-localized cytochrome P450 enzyme F3′H catalyzes the stereospecific 3′-hydroxylation of the flavonoid B-ring, thereby directing metabolic flux toward cyanidin biosynthesis [4].

Within plants, R2R3-MYB transcription factors (TFs) serve as major controllers of anthocyanin biosynthesis. Illustrative examples include BrMYB2 in *Brassica rapa* and ZmMYB31 in *Zea mays* activating *F3′H* to promote pigmentation, contrasted by the repressive function of CmMYB8 in *Chrysanthemum morifolium*, which suppresses *CmF3′H* expression [5–7]. Higher-order transcriptional networks involve upstream activators such as MdWRKY75 (*Malus domestica*) and VvWRKY26 (*Vitis vinifera*), which physically interact with MYB promoter regions to potentiate transcription [8, 9]. Conversely, ethylene response factors (ERFs) including ERF105 (*Pyrus pyrifolia*) and VvERF4 (*V. vinifera*) act as negative regulators by antagonizing MYB function or promoting histone deacetylation [10, 11]. Collectively, these findings establish R2R3-MYBs as the central hub orchestrating anthocyanin metabolic flux.

While substantial progress has been made in understanding *A. palmatum* leaf coloration through genetic, transcriptomic, genomic, and metabolic analyses—including the identification of key MYB TFs (e.g. *ApMYB1*) governing anthocyanin biosynthesis—a critical knowledge gap remains in the epigenetic regulation of its seasonal pigment transitions [12–17]. This particularly concerns chromatin regulatory processes involving 3D genome organization, dynamic chromatin accessibility, and DNA methylation patterns. Emerging epigenetic technologies have enabled unprecedented insights into chromatin regulation. Hi-C, a technique for mapping 3D chromatin interactions, has revealed dynamic chromatin architecture in plants, such as compartmentalization of active A compartments and repressive B compartments in *Arabidopsis*, rice, and poplar [18–24]. Complementing this, ATAC-seq identifies TF-binding accessible chromatin regions. In plants, it has been used to reveal cis-regulatory competition in apple anthocyanin/flavonol pathways and localize key regulatory elements in *Eucommia ulmoides* ‘Ziye’ anthocyanin biosynthesis [25, 26]. Cytosine DNA methylation dynamically regulates gene expression via CG, CHG, and CHH sites, influencing plant stress responses and pigmentation. Examples include light-induced hypomethylation promoting anthocyanin synthesis in pears, hypermethylation suppressing pigmentation in chrysanthemum (*CmMYB6*) and grape/apple (*VvMYBA1*/*MdMYB10*), and hypomethylation-driven white flesh mutants in radish (*RsMYB1*) [27–30]. Integrating these epigenetic layers—chromatin architecture, accessibility, and methylation—provides a comprehensive framework for decoding the molecular basis of plant trait plasticity and guiding precision breeding for ornamental traits.

‘Duocai’ is an excellent ornamental leaf cultivar of *A. palmatum*, exhibiting vibrant red foliage in spring that transitions to green in summer, before turning a brilliant red again in autumn. Our recent research has demonstrated that these seasonal color changes in ‘Duocai’ are closely linked to variations in anthocyanin content [15]. In this study, we utilized ‘Duocai’ as a model to explore its leaf color transition across different seasons, employing transcriptomic, metabolomic, Hi-C, ATAC-seq, and methylation sequencing technologies. The integrated multi-omics analysis identified the key TF, *ApMYB2*, along with its upstream positive regulator ApWRKY26 and negative regulator ApERF4, which collectively regulate the seasonal leaf color changes in ‘Duocai’. Functional assays delineated the molecular cascade whereby ApWRKY26 and ApERF4 dynamically modulate anthocyanin biosynthesis through *ApMYB2*-mediated transcriptional control. Our findings advance the understanding of seasonally differential anthocyanin accumulation mechanisms in *A. palmatum* ‘Duocai’, offering actionable insights for precision enhancement of foliage pigmentation in Acer species.

## Results

### Dynamics of anthocyanin content and transcriptomic reprogramming in *A. palmatum* leaf development

**Figure 1:**
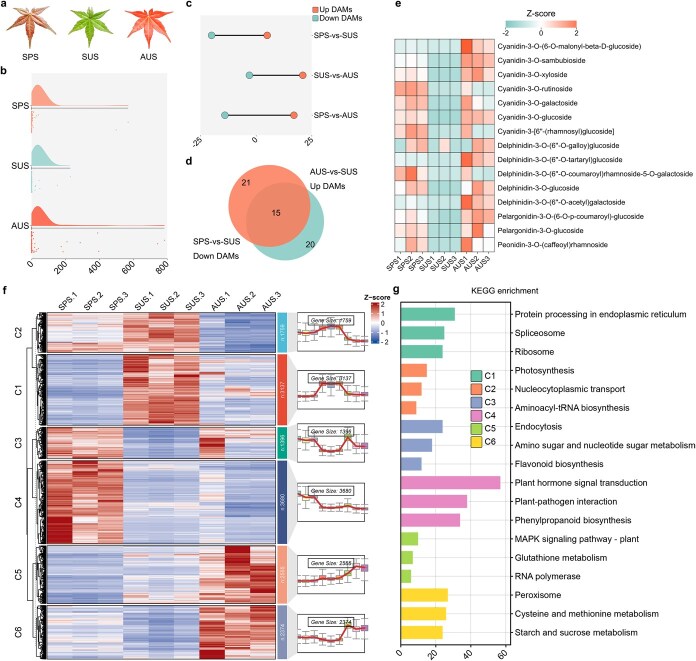
Seasonal pigmentation changes and transcriptome analysis of *A. palmatum* leaves. (a) Phenotypes of *A. palmatum* leaves: SPS, SUS, and AUS. (b) Rain cloud plot showing anthocyanin content in SPS, SUS, and AUS leaves. (c) Comparative analysis of differentially regulated metabolites across seasonal transitions (AUS vs SPS, SUS vs SPS, AUS vs SUS) in *A. palmatum*. (d) The Venn diagram illustrates the overlap among down DAMs in SPS-vs-SUS and up DAMs in SUS-vs-AUS comparisons. (e) Heatmap of anthocyanidin expression levels in leaves across different seasons. (f) Time-series clustering via HCA identifies six transcriptional modules (C1–C6) with distinct seasonal activation patterns. (g) KEGG enrichment analysis. The abscissa denotes the quantity of genes enriched in each KEGG pathway.

Throughout the cycle of leaf color change, the *A. palmatum* leaves undergo a transition from reddening in spring (SPS) to turning green in summer (SUS) and reddening again in autumn (AUS) (Fig. 1a). LC–MS/MS analysis identified 87 anthocyanin-related compounds, which were categorized into eight distinct groups (Table S1): cyanidins (24), delphinidins (23), peonidins (11), malvidins (8), petunidins (7), pelargonidins (6), proanthocyanidins (6), and flavonoids (2). Raincloud visualization revealed that anthocyanin concentrations peaked in SPS and AUS leaves (Fig. 1b). All the biological duplication demonstrated high reproducibility, confirming the robustness of metabolomic datasets (Fig. S1a). In both the spring and autumn leaves, anthocyanins were the dominant compounds at their respective stages. In summer leaves, the content of cyanidins and delphinidins was relatively similar (Fig. S1b). The relative abundance of cyanidin, delphinidin, peonidin, and pelargonidin metabolites in summer leaves exhibited a notable decline compared with that in spring leaves. As for malvidin and petunidin, their abundance was extremely low (<1 μg·g^−1^) in all three stages (Fig. S1c).

Significantly varying anthocyanins were obtained in leaves across different seasons of *A. palmatum* (Fig. 1c and Fig. S1d). Seasonal analyses demonstrated that anthocyanin accumulation peaked in autumnal foliar tissues, with spring and summer specimens exhibiting sequentially reduced concentrations. Comparative metabolomic analysis revealed 15 metabolites showing distinct accumulation patterns—depleted in SUS-vs-SPS comparisons but enriched in AUS-vs-SUS contrasts (Fig. 1d)—comprising 7 cyanidins, 5 delphinidins, 2 pelargonidins, and 1 peonidin (Fig. 1e). Of particular note, the cyanidin-3-O-glucoside content in SPS and AUS was several hundred times that of SUS. We hypothesize that these DAMs, particularly those enriched in the anthocyanin biosynthetic pathway, serve as critical regulators of seasonal pigmentation shifts in *A. palmatum*.

RNA-seq analysis investigated transcriptomic dynamics across three developmental phases to identify gene expression patterns underlying leaf color transitions. A total of 14 902 genes exhibited differential expression between sequential growth stages. The transition from spring to summer involved more down-regulated genes, while the transition from summer to fall was associated with more up-regulated genes. AUS leaves displayed balanced transcriptional activity, contrasting with spring’s skewed distribution (Table S2 and Fig. S2a). Hierarchical cluster analysis (HCA) of the transcriptome results revealed distinct expression patterns across the three seasons, identifying six major clusters of expression profiles (Fig. 1f). These clusters can be broadly categorized into the following groups: summer up-regulation (C1), autumn down-regulation (C2), summer down-regulation (C3), spring up-regulation (C4), spring down-regulation (C5), and autumn up-regulation (C6) (Fig. S2b and c). KEGG pathway analyses were conducted for each cluster to identify biological processes affected by specific seasons. Among these, the gene expression profile in C3 exhibited temporal congruence with anthocyanin accumulation dynamics during leaf development. KEGG pathway-based functional characterization of transcriptionally altered genes demonstrated their active participation in biosynthetic processes underlying flavonoid production (Fig. 1g).

### Chromatin features correlate with gene expression

Seasonal library preparation was implemented across spring, summer, and autumn growth phases, including nine ATAC-seq, nine Whole-genome bisulfite sequencing (WGBS), and six Hi-C replicates to profile chromatin dynamics. Similar to the transcriptome data, all epigenome datasets exhibited high Pearson correlation values, suggesting a high degree of reproducibility in our dataset (Fig. S3).

**Figure 2:**
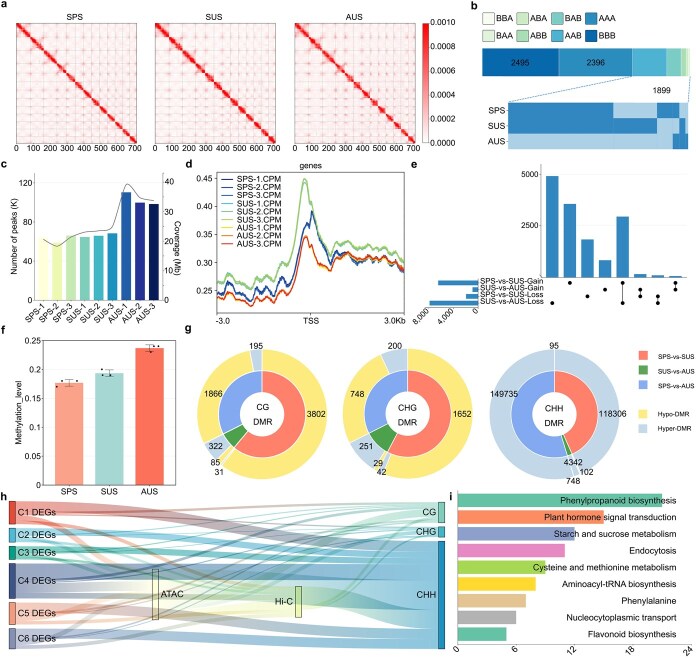
An epigenomic atlas of *A. palmatum* during leaf development in different seasons. (a) The high-resolution landscapes of chromatin organization. (b) Switches A/B compartment states during leaf development: A compartment and B compartment. (c) Peak numbers and peak coverage dynamics for ATAC-seq. (d) Read count profiles of accessible chromatin at the TSS for ATAC-seq. (e) The UpSet plots show gain (upper part) and loss (lower part) DARs between consecutive seasons. The ordinate indicates the count of DARs (intersection size) for single stages (single dots) and their combinations. (f) DNA methylation levels in SPS, SUS, and AUS across three contexts (CG, CHG, and CHH). Data are shown as mean ± SD from three biological replicates. (g) Pie chart indicating the number of hypomethylation and hypermethylation regions. (h) Sankey diagram showing DEGs regulated by 3D chromatin architecture, chromatin accessibility, and methylation. Hi-C represents DEGs located in at least one compartment, A or B. ATAC-seq and methylation represent DEGs with at least one corresponding peak. (i) KEGG enrichment analysis. The abscissa indicates the count of genes enriched in each KEGG pathway.

This X-shaped interaction architecture of the *A. palmatum* Hi-C map shows that it is consistent with the reported plant chromatin folding patterns (Fig. 2a). Chromatin compartment state transitions between A and B subtypes were systematically mapped across three developmental phases. Genome-scale analysis revealed that broad chromatin compartment dynamics remained predominantly stable (Fig. 2b). Compartment state transitions exhibited sequential patterning, where A to A to B conversions predominated, followed by B to A to B rearrangements. GO and KEGG analysis were assigned respectively (Fig. S4 and Table S3).

ATAC-seq reflected the dynamic changes in chromatin levels during the development of *A. palmatum* leaves. In total, 109 624, 106 793, and 156 501 peaks were obtained for the SPS, SUS, and AUS periods, respectively (Fig. 2c and Table S4). Across all samples, we detected a total of 194 631 accessible chromatin regions (ACRs), which showed transcription start site (TSS) enrichment (Fig. 2d). The dynamics of chromatin accessibility gain and loss exhibited seasonal variation. The transition from spring to summer in leaf coloration was characterized by the acquisition of numerous open chromatin regions (6462 DARs). In contrast, the shift from summer to autumn correlated with a substantial decrease in accessible genomic regions, with 7860 DARs being lost during this period (Fig. 2e and Table S5).

DNA methylation levels gradually increased with leaf development: the lowest in spring, higher in summer, and the highest in autumn (Fig. 2f and Table S6). A total of 123 982 DMRs (differential methylation regions) were identified between spring and summer samples, 5660 DMRs between summer and autumn samples, and 152 780 DMRs between spring and autumn samples. The CHG context had the lowest number of DMRs, while the CHH context had the highest, indicating differences in the diverse DNA methylation backgrounds of *A. palmatum* (Fig. 2g).

We also examined the relationship between gene expression and multiple chromatin characteristics. Our transcriptomic correlation study showed that changes in transcription are positively associated with chromatin structure and accessibility (Fig. S5a), while methylation is negatively correlated with transcription (Fig. S5b). Additionally, we mapped the methylation levels across the apex of open chromatin peaks, encompassing 1 kb of adjacent sequence on either side, and noted a consistent decrease in DNA methylation across all sequence contexts in proximity to these open chromatin peaks (Fig. S5c).

During the leaf color transition, 37% of DEGs exhibited variations in chromatin open regions, while 24% showed dynamic A/B compartment switching. Concerning methylation, 69% of DEGs displayed at least one methylation difference (Fig. 2h and Table S7). Notably, 1116 DEGs were impacted by both chromatin traits and methylation levels, significantly contributing to phenylpropanoid biosynthesis (Fig. 2i).

### Characterization of *ApMYB2* implicated in anthocyanin biosynthesis

**Figure 3:**
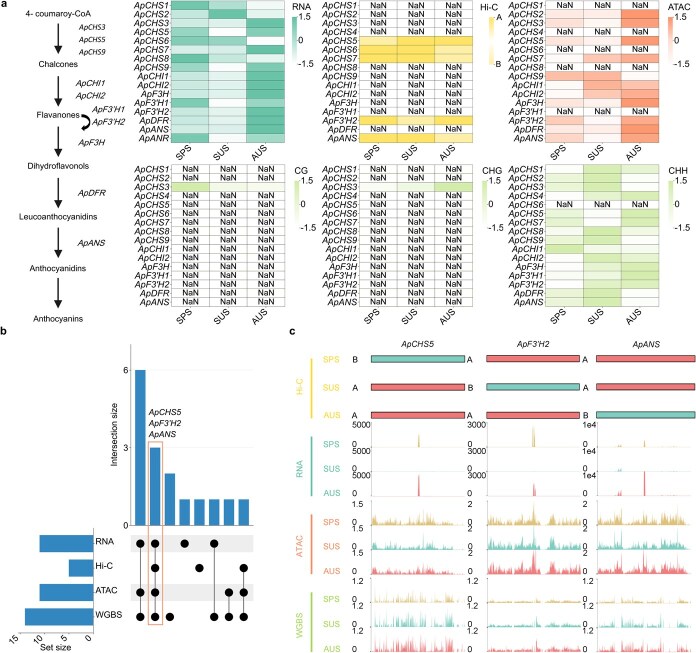
Transcriptional and epigenomic atlas during anthocyanin synthesis in *A. palmatum*. (a) Anthocyanin biosynthetic pathway. Heatmaps for RNA-seq, Hi-C, ATAC-seq, and WGBS data. RNA-seq, ATAC-seq, and WGBS data are presented as Z-scores; Hi-C data are labeled by A/B compartments. NaN (not a number) indicates missing values. (b) UpSet plots show transcriptional and epigenomic statistics for DEGs in the anthocyanin pathway. Y-axis shows the number of DEGs (size of crosses) for both transcriptional and epigenomic marks (single sites) and their combination. (c) Dynamic tracks of transcription (RNA) and epigenetic modifications (Hi-C, ATAC-seq, and WGBS) for key genes—*ApCHS5*, *ApF3'H2*, and *ApANS*.

To thoroughly analyze the synthesis pattern and material basis of anthocyanins throughout *A. palmatum*’s development, we concentrated on screening DEGs in this pathway (Fig. 3a). Three genes—*ApCHS5*, *ApF3'H2*, and *ApANS*—were found to align with anthocyanin trends and were influenced by chromatin dynamics. Therefore, we hypothesized that they might be critically involved in anthocyanin metabolism (Fig. 3b and c).

**Figure 4:**
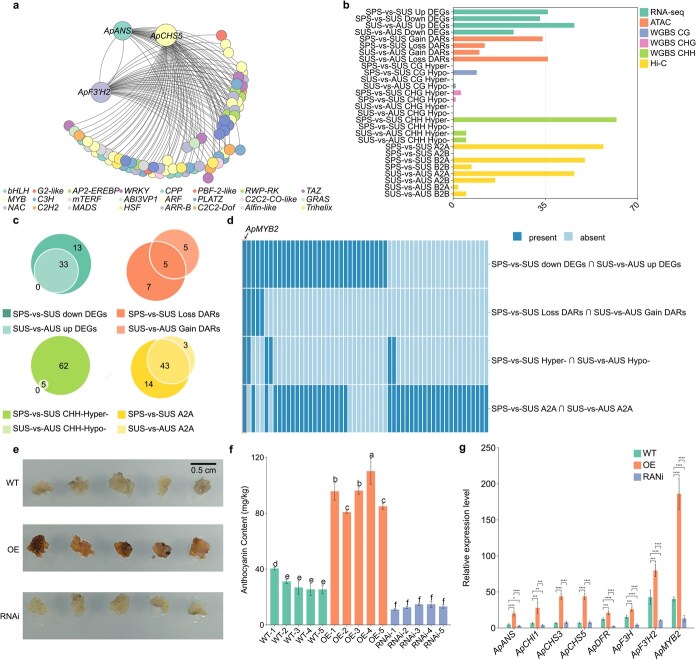
*ApMYB2* promotes anthocyanin synthesis in *A. palmatum.* (a) Network of TFs associated with the three *ApCHS5*, *ApF3'H2*, and *ApANS* genes constructed by integrating RNA-seq data. (b) Number of differentially expressed associated TFs, from (a), regulated by transcriptional and chromatin features. The horizontal coordinates represent the number of these TFs. (c) Venn diagram showing associated TFs with opposite expression patterns, chromatin accessibility, and methylation levels between spring-summer (SPS-vs-SUS) and summer-autumn (SUS-vs-AUS). Note: Since the intersection of A2B and B2A was not counted, we counted here the transcription factors that were located in A compartment in all three seasons. (d) Heat map of intersecting TFs in (c). The heatmap uses a binary indicator scheme: presence of an intersection (i.e. the TF is included in the overlapping set) and absence of an intersection (not included in the overlapping set) are labeled respectively. A highlighted box represents ApMYB2, a TF that is located in the active compartment throughout the growth cycle with expression levels much higher in SPS and AUS than in SUS, along with reduced chromatin accessibility and increased methylation in SUS. (e) The phenotype of transgenic callus overexpressing and silencing (OE and RNAi) ApMYB2. (f) Anthocyanin content in WT callus, the OE callus and RNAi callus. (g) Expression profiles of ApMYB2 and anthocyanin biosynthesis genes in WT, OE, and RNA interference (RNAi) callus lines. Values are reported as means ± standard deviation (*n* = 3). Error bars correspond to SD. Lowercase letters denote significant differences between groups. Asterisks indicate pairwise comparison significance thresholds.

To determine the seasonal dynamics of anthocyanin derivative allocation in *A. palmatum*, transcriptional networks involving three biosynthetic genes and various regulatory factors were comparatively profiled. The analysis revealed 69 TFs with strong correlation coefficients (|*R*| > 0.8) with anthocyanin synthesis genes (Fig. 4a). We then assessed these TFs’ chromatin accessibility, methylation levels, and chromatin compartments (Fig. S6a and Table S8).

To illustrate the possible regulation of anthocyanin biosynthesis-related gene expression by the transcriptome and epigenome, we focused on TFs with contrasting changes between spring-vs-summer (SPS-vs-SUS) and summer-vs-autumn (SUS-vs-AUS) (Fig. 4b). Using anthocyanin content changes as a reference, we conducted detailed transcriptomic and epigenomic analyses on these TFs. We identified 33 TFs down-regulated in the former and up-regulated in the latter, 5 TFs with decreased chromatin accessibility in the former and increased in the latter, 8 TFs with higher methylation levels in the former, and 43 TFs enriched in A compartment in both former and latter (Fig. 4c).

Notably, *ApMYB2* was in the active region throughout the growth cycle, with high expression in spring and autumn, lower in summer, reduced chromatin accessibility, and increased methylation in summer (Fig. 4d and Fig. S6b). Evolutionary analysis positioned *ApMYB2* within clade VI of the R2R3-MYB TF family, demonstrating closest phylogenetic affinity to Arabidopsis anthocyanin regulators (Fig. S6c). Therefore, *ApMYB2* is a promising candidate for studying its effect on anthocyanin synthesis.

We generated transgenic callus (OE for overexpression and RNAi for silencing) of *ApMYB2* to investigate its regulatory role in anthocyanin synthesis. Genomic DNA (gDNA) PCR verification confirmed the successful integration of the exogenous fragment into the callus genome, with positive bands specifically detected in OE and RNAi lines but not in wild-type (WT) callus (Fig. S7a and b). Compared to WT callus, the OE callus exhibited a redder color (Fig. 4e), higher anthocyanin content (Fig. 4f), and increased transcription of anthocyanin synthesis genes (Fig. 4g). Conversely, the RNAi callus showed the opposite trends. To further confirm the effect of *ApMYB2* on anthocyanin biosynthesis pathway genes, supplementary experiments were conducted in *A. palmatum* itself. In formal experiments, leaves were treated with the antisense oligonucleotide of *ApMYB2*, with water and sense oligonucleotide as controls (Fig. S8a). The qRT-PCR analysis revealed that silencing *ApMYB2* in *A. palmatum* led to reduced expression levels of anthocyanin biosynthesis genes (Fig. S8b–j). Ectopic OE of *ApMYB2* in poplar (Fig. S9a) further confirmed its role in anthocyanin synthesis. Transgenic poplar seedlings overexpressing *ApMYB2* exhibited increased anthocyanin content (Fig. S9b and c).

### ApMYB2 regulates the expression of *ApF3'H2*, which is essential for anthocyanin biosynthesis

**Figure 5:**
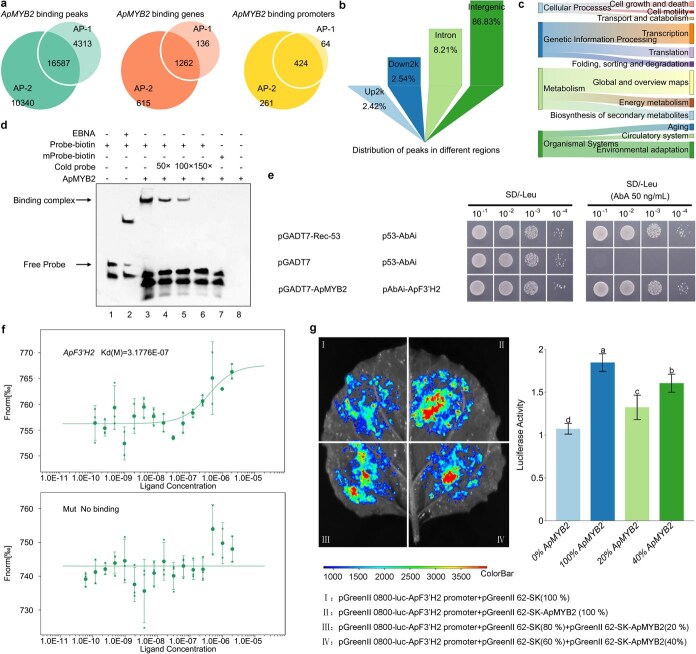
*ApMYB2* regulates anthocyanin synthesis genes *ApF3'H2*. (a) Overlap of ApMYB2 binding peaks/genes/promotes in two technical replicates of DAP-seq (AP1 and AP2). (b) Statistics of distribution regions of binding sites for ApMYB2. (c) Metabolic pathways enriched in genes containing ApMYB2-binding sites as detected by DAP-seq. (d) EMSA were performed with affinity-purified His-ApMYB2 fusion protein, which was incubated with biotin-labeled probes derived from the ApF3'H2 fragment—either in WT or mutated form. A cold probe served as a competitor, where ‘-’ indicates absence and ‘+’ denotes presence. (e) Yeast one-hybrid (Y1H) analysis confirmed ApMYB2-*ApF3'H2* promoter interaction. Assay detects interaction between ApMYB2 (prey, GAL4 activation domain-fused) and *ApF3'H2* promoter (bait, in pAbAi vector). Transformants were screened on SD/−Leu medium supplemented with 50 ng•ml^−1^ AbA, and the empty pGADT7 vector was used as the negative control. (f) Measuring interactions between ApMYB2 and the promoter of *ApF3'H2* using MST. The dissociation constant (Kd) of ApF3'H2 is about 3.1776E−07 M, while the mutant has no Kd. (g) ApMYB2 activates the *ApF3'H2* promoter in *N. benthamiana* leaves via dual-luciferase assays. Error bars correspond to SD. Lowercase letters denote significant differences between groups. Asterisks indicate pairwise comparison significance thresholds.

To further identify genes directly regulated by ApMYB2, DAP-seq was employed. A total of 31 240 binding peaks corresponding to 2013 genes were identified in genomic regions from two technical replicates of *ApMYB2* (Fig. 5a and Table S9). We organized the genomic regions containing the recognition sites and counted the frequency distribution of the ApMYB2-binding regions (Fig. 5b). Functional annotation using the KEGG pathway database was also analyzed (Fig. 5c). Specifically, DAP-seq analysis show that ApMYB2 can directly bind to *ApF3'H2*, thereby affecting the activity of anthocyanin compounds and their hydroxylation pattern.

EMSA analysis revealed that protein-DNA complex formation occurred when ApMYB2 was incubated with a labeled probe containing a fragment of the *ApF3'H2* promoter. Additionally, the mutant probe did not bind to the ApMYB2 protein (Fig. 5d). Subsequently, we assessed the interaction between ApMYB2 and the promoter of *ApF3'H2* using Y1H assays, with screening conducted on SD/-Leu selection plates supplemented with 50 ng•ml^−1^ AbA. The *ApF3'H2* promoter was cloned as bait in a reporter vector, with ApMYB2 as prey. Positive controls and bait/prey co-transformants exhibited growth on selective media (Fig. 5e and Fig. S10a), confirming the binding of ApMYB2 to the *ApF3'H2* promoter. EMSA and yeast one-hybrid (Y1H) confirmed this specific interaction *in vitro*, corroborating DAP-seq data. To quantify the affinity of ApMYB2 for *ApF3'H2*, we determined the binding dissociation constant (Kd) of ApMYB2 using microscale thermophoresis (MST). Fluorescently tagged ApMYB2 exhibited dose-dependent binding responses to the *ApF3'H2* promoter region (Kd = 3.18 × 10^−7^), while no specific interaction was observed in negative control assays (Fig. 5f). The data demonstrate that ApMYB2 interacts with the ApF3'H2 promoter. Furthermore, we infiltrated *Nicotiana benthamian* leaves with *Agrobacterium* mixtures containing two components: one harboring the *ApF3'H2* promoter-driven LUC reporter construct and the other carrying pGreenII 62-SK-ApMYB2 at different ratios relative to a GFP control. We observed that LUC activity progressively increased with the addition of pGreenII 62-SK-ApMYB2 protein. The findings confirm that ApMYB2 directly modulates the transcriptional activation activity of *ApF3'H2* (Fig. 5g). Collectively, these data underscore the critical function of *ApMYB2* in modulating *ApF3'H2* transcriptional activity during leaf development in *A. palmatum*.

### Dual regulation of *ApMYB2* by ApWRKY26 protein and ApERF4 protein in anthocyanin biosynthesis

**Figure 6:**
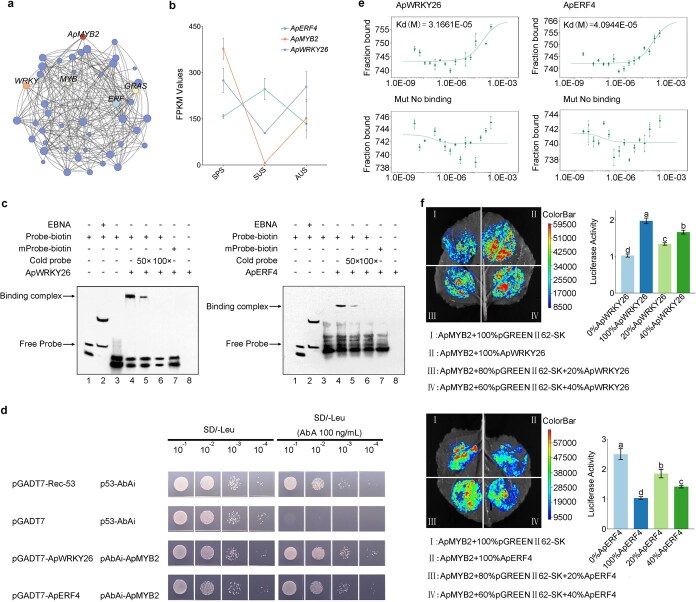
ApWRKY26 and ApERF4 dynamically regulate *ApMYB2*. (a) Gene network associated with the *ApMYB2* based on Y1H results. (b) Expression of *ApMYB2*, *ApWRKY26*, and *ApERF4* in leaves from spring, summer, or autumn. (c) EMSA assays with His-ApWRKY26 and His-ApERF4 fusion proteins incubated with biotin-labeled WT or mutated ApMYB2 probes. The cold probe served as a competitor. -, absence; +, presence. (d) Y1H assay showing ApWRKY26 and ApERF4 respectively binding to the promoter of *ApMYB2*. Assay detects interaction between ApWRKY26/ApERF4 (prey, GAL4 activation domain-fused) and the promoter of *ApMYB2* (bait, in pAbAi vector). Transformants were selected on SD/−Leu medium containing 50 ng•ml^−1^ AbA. (e) MST measurements of interactions between ApWRKY26/ApERF4 and the promoter of *ApMYB2* showed dissociation constants (Kd) of 3.17E^−^05 M for ApWRKY26 and 4.09E−05 M for ApERF4, while the mutant had no detectable Kd. The vertical coordinates represent the fraction bound, with no units (dimensionless, ranging from 0 to 1). (f) ApWRKY26/ApERF4 activates the promoter of *ApMYB2* in *N. benthamiana* leaves via dual-luciferase assays. Error bars correspond to SD. Lowercase letters denote significant differences between groups. Asterisks indicate pairwise comparison significance thresholds.

To further elucidate the TFs regulating *ApMYB2* and their impact on anthocyanin synthesis, we conducted yeast one-hybrid screening experiments. Well-grown and rescreened clones underwent colony PCR sequencing, resulting in 48 clones after BLAST comparison of the sequencing results. Correlation analysis between the transcriptome data of *ApMYB2* and the genes identified in these 48 clones revealed a positive correlation between *ApWRKY26* and *ApMYB2*, while *ApERF4* showed a negative correlation (Fig. 6a and Table S10). The expression profiles of *ApMYB2* and *ApWRKY26* matched the accumulation pattern of anthocyanins across various seasons, in contrast to *ApERF4*, which displayed a contrary tendency (Fig. 6b). Therefore, we tested whether ApWRKY26 and ApERF4 could play a regulatory role in the transcriptional activity of *ApMYB2*. EMSA analysis showed that both ApWRKY26 and ApERF4 were able to bind to the promoter of *ApMYB2* (Fig. 6c). The Y1H results further supported the conclusion that both ApWRKY26 and ApERF4 can activate the *ApMYB2* promoter—evidenced by their growth on SD/-Leu selection plates supplemented with 100 ng•ml^−1^ AbA(Fig. 6d and Fig. S10b). The MST results demonstrated that the dissociation coefficients of ApWRKY26 and ApERF4 were Kd (M) = 3.1661E−05 and Kd (M) = 4.0944E−05, respectively (Fig. 6e). In order to understand the regulatory role *ApWRKY26* and *ApERF4* on *ApMYB2*, we performed a dual-luciferase assays in *N. benthamiana* leaves. The results revealed dose-dependent transcriptional regulation: co-expression of *ApWRKY26* with the *ApMYB2* promoter-LUC construct enhanced bioluminescence, whereas *ApERF4* suppressed LUC activity. These antagonistic effects demonstrate *ApWRKY26*’s transactivation capacity versus *ApERF4*’s repressive function on *ApMYB2* transcription, mechanistically validated through promoter-proximal TF interplay (Fig. 6f).

**Figure 7:**
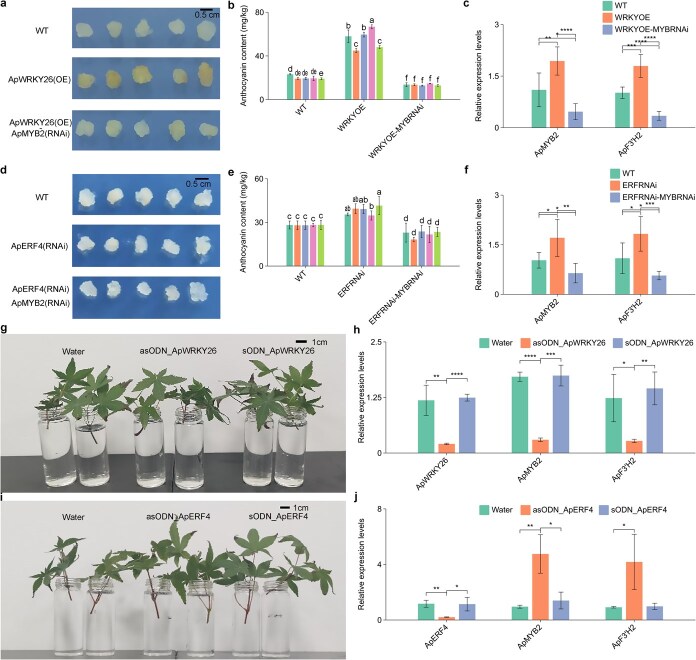
Regulatory effects of *ApWRKY26* and *ApERF4* on *ApMYB2*-mediated anthocyanin biosynthesis in *A. palmatum*. (a) Schematic representation of transgenic callus lines: WT callus, *ApWRKY26-*overexpressing (*ApWRKY26*-OE), and *ApWRKY26*-overexpressing with *ApMYB2* co-silenced (*ApWRKY26*-OE + *ApMYB2*-RNAi). (b) Anthocyanin content in WT, *ApWRKY26*-OE, and *ApWRKY26*-OE + *ApMYB2*-RNAi callus. (c) Relative expression levels of ApMYB2 in the three callus lines. (d) Schematic representation of transgenic callus lines: *ApERF4*-silenced (*ApERF4*-RNAi), *ApERF4*-silenced with *ApMYB2* co-silenced (*ApERF4*-RNAi + ApMYB2-RNAi), and WT callus. (e) Anthocyanin content in *ApERF4*-RNAi, WT, and *ApERF4*-RNAi + *ApMYB2*-RNAi callus. (f) Relative expression levels of *ApMYB2* in the three callus lines. (g) Phenotypic comparison of *A. palmatum* leaves treated with water (control), antisense oligonucleotide targeting *ApWRKY26* (asODN_*ApWRKY26*) and sense oligonucleotide (sODN_*ApWRKY26*). (h) Relative expression levels of *ApMYB2* and anthocyanin biosynthesis genes in leaves from (g). (i) Phenotypic comparison of *A. palmatum* leaves treated with water (control), antisense oligonucleotide targeting *ApERF4* (asODN_*ApERF4*) and sense oligonucleotide (sODN_*ApERF4*). (j) Relative expression levels of *ApMYB2* and anthocyanin biosynthesis genes in leaves from (i). Values are reported as means ± standard deviation (*n* = 3). Error bars correspond to SD. Lowercase letters denote significant differences between groups. Asterisks indicate pairwise comparison significance thresholds.

To further understand the regulatory mechanisms of ApWRKY26 on *ApMYB2*, we generated transgenic callus of *A. palmatum* overexpressing *ApWRKY26*. To clarify the genetic relationship between the two, we additionally constructed transgenic lines where *ApMYB2* was co-silenced in the *ApWRKY26*-overexpressing background (*ApWRKY26*-OE + *ApMYB2*-RNAi), with gel electrophoresis confirming successful transgene integration (Fig. 7a and Fig. S7c–e). Quantitative analysis showed that anthocyanin accumulation and *ApMYB2* expression were most prominent in the *ApWRKY26* OE callus, followed by the WT callus, while the lowest levels were observed in the *ApWRKY26*-OE + *ApMYB2*-RNAi callus (Fig. 7b and c). Notably, the enhanced anthocyanin phenotype induced by *ApWRKY26* OE was eliminated by co-silencing *ApMYB2*, with the content even lower than that of the WT, confirming that ApWRKY26 promotes anthocyanin biosynthesis through *ApMYB2*, which acts downstream.

In parallel, to elucidate the regulatory mechanism of ApERF4 on *ApMYB2*, we generated *A. palmatum* transgenic callus lines with *ApERF*4 silenced (Fig. 7d). We also constructed lines where *ApMYB2* was co-silenced in the *ApERF4*-silenced background (*ApERF4*-RNAi + *ApMYB2*-RNAi) to explore their genetic dependency, with gel electrophoresis confirming successful transgene integration (Fig. 7d and Fig. S7f, g). The *ApERF4*-silenced callus exhibited increased anthocyanin content and *ApMYB2* expression, while the *ApERF4*-RNAi + *ApMYB2*-RNAi callus showed reduced anthocyanin levels compared to both the *ApERF4*-silenced callus and the WT callus (Fig. 7e and f). This reversal indicates that the increased anthocyanin accumulation caused by *ApERF4* silencing relies on the presence of functional *ApMYB2*. These findings imply that ApERF4 negatively regulates *ApMYB2* transcription, thereby weakening its role in promoting anthocyanin synthesis.

To validate these regulatory interactions in *A. palmatum*, we performed in planta experiments targeting *ApWRKY26* and *ApERF4* (Fig. 7g and i). Leaves treated with an antisense oligonucleotide against *ApWRKY26* exhibited reduced *ApMYB2* expression and decreased transcription of anthocyanin biosynthesis genes compared to water and sense oligonucleotide controls (Fig. 7h). Conversely, silencing *ApERF4* using an antisense oligonucleotide increased *ApMYB2* expression and upregulated anthocyanin pathway genes (Fig. 7j). These trends mirrored the transgenic callus results, confirming that ApWRKY26 promotes, and ApERF4 inhibits anthocyanin biosynthesis through transcriptional regulation of *ApMYB2*.

## Discussion

Anthocyanins, which are extensively distributed in angiosperms, exhibit significant seasonal variations in their content across plants. However, little is known about the seasonal transition of leaf color in maples such as *A. palmatum*. To address this gap, we used *A. palmatum* ‘Duocai’ as our study object and generated probably the first set of transcriptomic and epigenomic data during the seasonal shift of leaf color in *A. palmatum*. Multi-omics integration of metabolomic, RNA-Seq, ATAC-Seq, Hi-C, and WGBS datasets across three developmental phases (SPS, SUS, AUS) established a framework to decode seasonal anthocyanin dynamics and advance precision enhancement of foliage pigmentation in Aceraceous species.

### Key metabolites in the seasonal transformation

In this study, cyanidins, delphinidins, pelargonidins, and peonidins metabolites exhibited a greater decrease than increase in the SPS_vs_SUS, and increased much more than decreased in the SUS_vs_AUS. In particular, cyanidin-3-O-glucoside (C3G) content was several hundred times higher in SPS and AUS than in SUS, suggesting that the accumulation of such metabolites is highly correlated with the reddening of *A. palmatum* leaves (Fig. 1).

A comparable phenomenon was noted in *Phoebe bournei*, where the anthocyanin level in crimson leaves exceeded that in verdant ones, and there was a notable decline in anthocyanin concentration as the crimson leaves aged. Notably, C3G proved to be the most prominent anthocyanin present in the red leaves, hinting at its pivotal role in the alteration of leaf hue [31]. Similarly, in *Toona sinensis*, research revealed that cultivars with vibrant red foliage possess significantly elevated levels of C3G compared to their green-leaved counterparts [32]. In cotton, the petals of PF (exhibiting pink petals) and Rs (exhibiting red petals) showed a high accumulation of C3G, as opposed to X74 (exhibiting white petals) [33].

More importantly, relevant studies on Acer species further support this conclusion. Previous research on various common Acer species has extensively examined anthocyanin content changes: in *A. pseudosieboldianum*, C3G remains at high levels throughout leaf development and is recognized as a main coloring substance [34]; and in *A. mandshuricum*, it is the predominant metabolite in anthocyanin biosynthesis and significantly correlates with color formation [35]. For *Acer circumlobatum*, *A. ornatum* Carr. var. matsumurae, and 119 surveyed Acer taxa, C3G is identified as the major anthocyanin in both autumn-colored and spring-sprouted leaves [36]. C3G remains the predominant anthocyanin in *A. palmatum* Thunb and its cultivars, such as *A. p*. var palmatum, *A. p*. ‘Semi K2’, *A. p*. ‘Dissectum rubrifolium’, *A. p.* ‘Coreanum’, *A. p.* ‘Dissectum nigrum’, and *A. p.* ‘Bloodgood’ [37]. Additionally, in red leaves of *Acer pictum* subsp. Mono, C3G is a stable anthocyanin with significantly upregulated content [38].

Taken together, these findings from both other plant genera and multiple Acer species strongly suggest that C3G is the most critical component contributing to leaf reddening during the seasonal transition of *A. palmatum* leaves, including the ‘Duocai’ cultivar.

### Integrated epigenomic-transcriptomic landscape finds key genes for anthocyanin synthesis

Eukaryotic organisms organize their genetic material by compacting it within the nucleus through complex 3D chromatin architectures, and these spatial arrangements are broadly classified into distinct compartments labeled A and B. Earlier research indicates that genes situated in chromosomal regions transitioning from compartment A to B are frequently associated with reduced transcriptional activity. Conversely, genes within zones shifting from B to A often display heightened expression levels [39, 40]. Chromatin in transcriptionally active regions of eukaryotic genomes is relatively loosely structured and susceptible to binding by regulatory proteins [41]. Chromatin accessibility is highly correlated with transcript levels during callus induction in wheat [42]. DNA methylation and demethylation enhance genomic plasticity [43]. The process typically involves the methylation of promoter areas, which inhibits the attachment of transcriptional activators, whereas it simultaneously promotes the binding of transcriptional repressors. This results in a decrease or cessation of transcriptional activity [44].

Our multi-omics integration (RNA-Seq, Hi-C, ATAC-Seq, and DNA methylation analyses) resolves a critical knowledge gap by mechanistically linking chromatin spatial dynamics to seasonal anthocyanin regulation in *A. palmatum* (Fig. 2). Most genes in *A. palmatum* anthocyanin synthesis show seasonal variability, co-regulated by chromatin accessibility, topology, and methylation (Fig. 3). Among them, changes in the expression of three key genes—*ApCHS5*, *ApF3'H2*, *ApANS*—were found to follow the same trend as anthocyanin synthesis and were regulated by all three epigenetic factors simultaneously. In particular, the *ApF3'H2* gene, which is in the A compartment with higher chromatin accessibility during the red leaf stage (spring and autumn), is more methylated during the green leaf period (summer). F3'H is a key nodal enzyme for cyanidin production [45]. Nitarska *et al*. obtained an *F3'H* knockout line in the red flowering poinsettia, which showed a drastic decrease in cyanidin content compared to the WT [46]. Functional studies in *Fagopyrum tataricum* demonstrate *FtF3H1*’s role as a metabolic gatekeeper enhancing anthocyanin accumulation in transgenics [47]. Comparative analyses suggest seasonal expression variation of *ApF3'H2* in *A. palmatum* during the different seasons resulted in large differences in the content of cyanidin.

### ApWRKY26 positively regulates *ApMYB2*-*ApF3'H2*-mediated anthocyanin synthesis, whereas ApERF4 negatively regulates this process

Several TFs, such as MYB, bHLH, NAC, PHR, and NF-Y, are positive regulators of *F3'H* expression [4, 48–51]. In particular, the regulatory role of MYB on *F3'H* has been demonstrated in a considerable number of species. In strawberry plants, FaMYB5 exerts a positive regulatory effect on anthocyanin accumulation via the trans-activation of *F3'H* [52]. Heterologous expression of maize ZmMYB31 in *Arabidopsis* alters flavonoid metabolism through *F3'H* activation [6]. The *F3'H* gene exhibited the highest upregulation in transgenic *Arabidopsis* harboring *AgMYB2* [53]. McMYB10 transcriptionally activates *McF3'H*, increasing anthocyanin production in crabapple [54].

Here, our results showed that OE of *ApMYB2* could activate the transcription of *ApF3'H2* in *A. palmatum*, leading to an increase in anthocyanins synthesis (Fig. 4). Experiments such as Y1H, LUC, EMSA, and MST revealed that ApMYB2 can directly activate *ApF3'H2* transcription. These findings suggest that the ApMYB2-*ApF3'H2* transcriptional module functions as a regulatory pathway driving anthocyanin accumulation in *A. palmatum* (Fig. 5).

In addition to MYB, it was found that other TFs, such as WRKYs, can also regulate anthocyanin biosynthesis. VvWRKY5 could positively regulate grapevine damage-induced anthocyanin accumulation by promoting jasmonic acid biosynthesis through interaction with VvMYBA1 [28]. It has been demonstrated that MdWRKY75 interacts with the promoter region of *MdMYB1*, thereby boosting pigment accumulation in apple fruits [8]. The BT2-WRKY40-MYB1 regulatory module has been shown to play a key role in fruit anthocyanin biosynthesis [55]. Similarly, MdWRKY10 could enhance its transcription by binding to the promoter of *MdMYB10*, which in turn enhances anthocyanin synthesis *in vivo* [56]. In pears, PyWRKY26 and PybHLH3 can jointly target the promoter of *PyMYB114* to enhance anthocyanin accumulation [57]. In grapevines, VvWRKY26 potentiates the *VvMYB5a*/*b*-mediated expression of genes associated with flavonoid biosynthesis [9].

Studies have shown that ERF TFs are indispensable for orchestrating anthocyanin biosynthesis. The TF ERF105 can activate the TF *MYB140*, which inhibits red pear anthocyanin accumulation [10]. VvERF4 binds to the promoter region of *VvMYB5a* and functions as a transcriptional repressor, facilitating deacetylation that decreases anthocyanin accumulation during grape maturation and hinders fruit color development [11]. Additionally, in pear species (Pyrus spp.), the PpERF9-PpTPL1 complex induces histone deacetylation through PpMYB114, thereby impeding the biosynthesis of anthocyanins [58]. Here, our results indicate that ApWRKY26 is a direct positive regulator of *ApMYB2*, while ApERF4 could negatively regulate *ApMYB2* transcription (Figs 6 and 7).

We proposed a model to elucidate the molecular mechanism by which ApWRKY26 and ApERF4 dynamically regulate *ApMYB2*-*ApF3'H2* in different seasons, affecting anthocyanin accumulation. In spring and autumn, increased expression of *ApWRKY26* promotes the transcriptional activation of *ApMYB2*, affecting the expression of *ApF3'H2* and positively regulating anthocyanin accumulation. Conversely, high expression of *ApERF4* during summer inhibits the transcriptional activation of *ApMYB2*, impacting the expression of *ApF3'H2* and negatively regulating anthocyanin accumulation.

In summary, based on metabolomic data, we identified cyanidin-3-O-glucoside as the key substance for seasonal leaf color shifts in *A. palmatum*. By integrating epigenomic-transcriptomic multi-omics data, we identified *ApF3'H2* and its upstream positive regulator *ApMYB2* as key genes affecting anthocyanin accumulation. ApWRKY26 and ApERF4 either promote or interfere with the transcriptional activation of *ApMYB2* across different seasons, dynamically regulating anthocyanin accumulation. The alternation of regulatory modules—comprising *ApWRKY26*-*ApMYB2*-*ApF3'H2* in spring and autumn and *ApERF4*-*ApMYB2*-*ApF3'H2* in summer—ensures anthocyanin accumulation and subsequently affects leaf coloration in *A. palmatum*.

## Materials and methods

### Plant materials

*Acer palmatum* ‘Duocai’ plants were sourced from the experimental fields in Hefei City, Anhui Province, China (N 31.86, E 117.27). Eight-year-old, uniformly sized saplings were cultivated under natural conditions in a subtropical monsoon climate, with regular irrigation. We gathered foliage from three seasonal stages for examination, each defined by distinct developmental phenotypes: spring (April 14; Stage S1, vibrant red pigmentation), summer (August 15; Stage S2, transitioning from red to green), and autumn (November 28; Stage S3, red coloration due to anthocyanin accumulation). For each stage, leaves were harvested from the same individual plants between 9:00 and 11:00 a.m. to minimize diurnal variation, then promptly frozen in liquid nitrogen and stored at −80°C for subsequent analysis.

### LC–MS/MS analysis of *A. palmatum* leaves

The LC–MS/MS conditions were as described in the method of Ni *et al*. based on the AB Sciex QTRAP 6500 LC–MS/MS platform [59]. A total of three groups of samples were selected, comprising nine samples in total. Samples were lyophilized under vacuum, ground into fine powder using a ball mill (MM400, Retsch) at 30 Hz for 1.5 min, and 50 mg of the powder was extracted with 500 μl of 50% methanol aqueous solution containing 0.1% hydrochloric acid. The mixture was vortexed for 5 min, sonicated for 5 min, and centrifuged at 12000 × *g* for 3 min at 4°C. The supernatant was collected, and the extraction was repeated once; the combined supernatants were filtered through a 0.22-μm microporous membrane prior to analysis. Chromatographic separation was performed on an ExionLC™ AD UPLC system equipped with a Waters ACQUITY BEH C18 column (1.7 μm, 2.1 mm × 100 mm) using a binary mobile phase consisting of 0.1% formic acid in water (Phase A) and 0.1% formic acid in methanol (Phase B). The gradient elution program was as follows: 5% B at 0 min, linearly increased to 50% B at 6 min, further increased to 95% B at 12 min and held for 2 min, then returned to 5% B at 14 min and held for 2 min for column re-equilibration, with a constant flow rate of 0.35 ml/min, column temperature maintained at 40°C, and an injection volume of 2 μl. Mass spectrometric detection was carried out on a QTRAP® 6500+ system operated in positive electrospray ionization mode with the ion source temperature set at 550°C, spray voltage at 5.5 kV, and curtain gas at 35 psi. Scheduled multiple reaction monitoring was employed with optimized declustering potentials and collision energies. Compound identification was achieved by matching against the in-house Metware Database, while quantification was performed using external calibration curves constructed with 14 concentration levels ranging from 0.01 to 5000 ng·ml^−1^. The analyte content in samples was calculated using the formula: content (μg·g^−1^) = (*c* × *V*)/(100 000 × *m*), where c represents the concentration (ng·ml^−1^) determined from the calibration curve, *V* is the extraction volume (μl), and *m* denotes the sample mass (g). To ensure data quality, a quality control sample (mixed standard solution) was analyzed after every 10 experimental samples, and system stability was verified by total ion chromatogram overlap. All data were acquired using Analyst 1.6.3 software and processed with MultiQuant 3.0.3 for quantitative analysis.

### RNA extraction, quantification, and quality control

Total RNA was isolated from 100 mg of flash-frozen leaves using the TRIzol Reagent (Invitrogen, CA, USA). Samples were homogenized in 1 ml of TRIzol reagent, incubated for 5 min at room temperature, and centrifuged at 12000 × g for 10 min at 4°C to remove debris. The supernatant was mixed with 200 μl of chloroform, vortexed vigorously, and centrifuged again under the same conditions. The aqueous phase was transferred to a new tube, and RNA was precipitated by adding 500 μl of isopropanol, followed by incubation at −20°C for 30 min and centrifugation at 12000 × g for 10 min at 4°C. The RNA pellet was washed twice with 75% ethanol, air-dried, and resuspended in 20 μl of RNase-free water. RNA purity was assessed by measuring absorbance ratios using a NanoDrop 2000 Spectrophotometer (Thermo Fisher Scientific, DE, USA). RNA integrity was evaluated using a Bioanalyzer 2100 System (Agilent Technologies, CA, USA). Additionally, RNA samples were electrophoresed on a 1.5% agarose gel.

### DNA extraction, quantification, and quality control

Genomic DNA was extracted from 100 mg of fresh plant tissue using a modified CTAB method. Briefly, samples were ground in liquid nitrogen and homogenized in 1 ml of CTAB buffer (2% CTAB, 1.4 M NaCl, 100 mM Tris–HCl pH 8.0, 20 mM EDTA, 1% PVP, 0.2% β-mercaptoethanol) at 65°C for 30 min. After adding chloroform:isoamyl alcohol (24:1), the mixture was centrifuged at 12000 × g for 10 min. The aqueous phase was collected, and DNA was precipitated with 0.6 volumes of isopropanol at −20°C for 1 h. The DNA pellet was washed with 70% ethanol, air-dried, and dissolved in 50 μl of AE buffer. DNA quality was verified by 0.8% agarose gel electrophoresis, and concentration was measured using a Qubit® 3.0 Fluorometer (Thermo Fisher Scientific) with the dsDNA HS Assay Kit. All samples were adjusted to 100 ng·μl^−1^ for downstream applications.

### RNA transcriptome sequencing

High-throughput transcriptome profiling was performed using the MGI-SEQ 2000 platform (Frasergen Bioinformatics, Wuhan, China). mRNA was enriched with oligo(dT) magnetic beads, and sequencing libraries were constructed using the VAHTS Universal V6 RNA-seq Library Kit for MGI, validated by Qubit 3.0 and Bioanalyzer 2100. Raw sequencing data were filtered by SOAPnuke to remove adapter-contaminated, low-quality, and high-N reads. Clean reads were aligned to the reference genome/transcripts using HISAT2 and Bowtie2, followed by transcript quantification via RSEM to calculate FPKM values [60–62]. Differential expression analysis was conducted using DESeq2 (v1.22.2) with replicates or edgeR without replicates, setting thresholds at FDR < 0.05 and |log₂FC| > 1 to identify significant DEGs [63]. Functional annotation of transcripts was performed using KEGG and GO databases, and enrichment analyses were conducted via hypergeometric tests and KOBAS 3.0. KEGG pathways and GO terms with *Q*-values ≤ 0.05 were respectively defined as significantly enriched pathways and functional entries. To validate the RNA-Seq data, we randomly selected nine DEGs and performed qRT-PCR analysis. The qRT-PCR results (Fig. S11) showed high consistency with the RNA-Seq data, validating the reliability of the transcriptomic profiling.

### Whole-genome bisulfite sequencing

Genomic DNA isolation involved CleanNGS-R paramagnetic beads (Vdo Biotech, China) and subjected to ultrasound treatment to break the DNA into fragments of 200–500 bp. Subsequently, end repair, 5′- phosphorylation, and 3′-dA tailing were performed and connected to a 5-methylcytosine modified adapter. DNA bisulfite conversion was achieved using the ZYMO EZ DNA Methylation-Gold Kit (Zymo Research), with subsequent gel cutting and PCR amplification. Paired-end sequencing (150 bp) was conducted on the Illumina NovaSeq 6000 platform. Quality control involved Trimmomatic trimming (adapter trimming, base quality filtering) and FastQC validation, yielding high-fidelity reads [64]. Bismark v0.22.3 aligned cleaned reads to the reference genome [65]. Differential methylation analysis identified DMRs via metilene v0.2–8 [66]. ChIPseeker annotated DMRs to promoter regions, with GO and KEGG pathway enrichment.

Subsequent methylation PCR detection is performed, with specific steps as follows: In a PCR tube, mix 20 μl sample with 5 μl Buffer MD, centrifuge briefly, and incubate at 37°C for 15 min; then add 100 μl Conversion Solution CR, mix, centrifuge briefly, and incubate at 54°C for 90 min (hold at 4°C if the next step is delayed). Add 200 μl Column Equilibration Buffer PS to the adsorption column in a collection tube, centrifuge at 12 000 rpm for 1 min, discard the waste, and return the column. Add 600 μl Buffer CL, transfer the product from the previous step to the column, invert 3–5 times, stand at room temperature for 10 min, then centrifuge at 12 000 rpm for 1 min, discard the waste, and return the column. Add 500 μl Rinse Buffer 1 (with anhydrous ethanol added) and centrifuge at 12 000 rpm for 1 min without discarding the waste; then add 200 μl Buffer DB, stand for 10–15 min, centrifuge at 12 000 rpm for 1 min, discard the waste, and return the column. Add 500 μl Rinse Buffer 2 (with anhydrous ethanol added) and centrifuge at 12 000 rpm for 1 min without discarding the waste; then add 200 μl Wash Buffer WB (with anhydrous ethanol added), centrifuge at 12 000 rpm for 1 min, discard the waste, and return the column. Centrifuge the column with the tube at 12 000 rpm for 2 min, transfer the column to a new tube, air dry for 3 min, add 20 μl Elution Buffer to the column membrane, incubate for 2 min, and centrifuge at 12 000 rpm for 1 min to collect DNA (preheating the elution buffer can improve recovery). The purified product is subjected to PCR with corresponding primers (Table S11), and then the PCR product is sequenced to compare and analyze the methylation status. The five gene fragments exhibit varying degrees of methylation, as verified by bisulfite sequencing (Table S12).

### ATAC-seq analysis

The sequence libraries were constructed based on previous studies [67]. After the samples were thoroughly ground in liquid nitrogen, lysate was added to cleave the cell membrane to obtain nuclei. Then, Tn5 transposase was added for enzymatic cleavage of open region chromatin DNA. PCR amplification generated indexed libraries, which were sequenced on an Illumina NovaSeq platform (San Diego, CA) with 150-bp paired-end reads (San Diego, CA, United States). Raw data processing involved Trimmomatic trimming (adapter removal, base quality filtering) and FastQC validation to generate high-fidelity reads [64]. Bowtie2 aligned clean reads to the reference genome, followed by Samtools/Picard processing to retain uniquely mapped reads [60]. MACS3 (v3.0.0a6) identified significant peaks in valid datasets [68]. DeepTools visualized TSS-associated signal intensities, while ChIPseeker annotated peaks to promoter regions [69, 70]. DARs was identified by DiffBind analysis (|log₂FC| ≥ 0.58, *P*-value ≤ .01) [71].

### Hi-C experiments

Hi-C library construction followed established protocols [72]. Cross-linking was performed using 3% formaldehyde (30 min, 4°C), followed by MboI (100 U, NEB) digestion and biotin-14-dCTP labeling (Invitrogen). Ligation with T4 DNA ligase (50 U, NEB) facilitated loop formation, supplemented by proteolytic cross-linking. Sonication sheared DNA into 300–500 bp fragments, which underwent end repair, A-tailing, adapter ligation, biotin pull-down, and PCR amplification. Quantified Hi-C libraries were sequenced on the MGI-seq platform (BGI, Shenzhen, China).

### Phylogenetic analysis and network construction

Reference sequences for the *Arabidopsis thaliana* MYB gene cluster were sourced from TAIR, providing the orthologous framework for candidate gene identification in *A. palmatum* [73]. Tbtools BLAST batch processing enabled initial retrieval of *A. palmatum* MYB candidates through bulk sequence alignment against the reference database [74]. HMMER3 analysis (v3.3.1) identified conserved MYB domains (PF00249) via *de novo* motif scanning of the *A. palmatum* genome [75]. We then performed a comprehensive motif analysis of the obtained sequences using the MEME [76]. Alignments was performed using MAFFT [77]. Low-quality alignment artifacts and spurious sequences were filtered out via trimAl (v1.2) [78]. Phylogenetic inference was conducted using FastTree under the JTT substitution mode [79]. Tree topology visualization was implemented in Chiplot website [80].

### Construction of recombination vectors and generation of transgenic plant materials

Vector Construction: The OE vectors for *ApMYB2* and *ApWRKY26* were constructed by cloning their respective coding sequences into the plant binary vector PCambia2300-35S under the control of the CaMV 35S promoter, generating pCambia2300-35S::*ApMYB2* and pCambia2300-35S::*ApWRKY26*. For RNAi vectors targeting *ApMYB2*, two separate constructs were generated: a single-gene silencing vector (pCambia2300-35S::RNAi-*ApMYB2*) was created by sequentially inserting its target fragment into pKANNIBAL using *Eco*RI/*Kpn*I and *Bam*HI/*Xba*I, followed by excision with *Sac*I/*Spe*I and transfer to pCambia2300 for kanamycin selection; a second vector (pCambia1300-35S::RNAi-*ApMYB2*) with hygromycin resistance was constructed using identical cloning steps but transferred to pCambia1300 instead, specifically designed for co-transformation with *ApWRKY26* OE lines. The dual-gene silencing vector targeting *ApERF4* and *ApMYB2* was constructed by synthesizing a tandem fragment of their first 200 bp and its reverse complement, cloning into pKANNIBAL, and transferring the hairpin to pCambia2300-35S. All constructs were verified by sequencing.

Plant Transformation: *A. palmatum* callus transformation was performed following a modified protocol. Briefly, 5-mm-long stem nodes from sterile seedlings were excised under aseptic conditions and pre-cultured on MP medium (MP medium: MS medium +30 g·l^−1^ sucrose +7 g·l^−1^ agar +1 mg·l^−1^ 6-BA +2 mg·l^−1^ 2, 4-D + 0.5 mg·l^−1^ NAA, pH = 5.8) for 3–4 days under a 16-h light/8-h dark photoperiod. Pre-cultured explants were immersed in an *Agrobacterium tumefaciens* suspension harboring the target vector, incubated with gentle shaking (20–30 min), and transferred to co-culture medium (MC) for 3–4 days in the dark at 25–28°C. Following co-cultivation, explants were washed 3–5 times with sterile water containing 500 mg·L^−1^ timentin (to eliminate *Agrobacterium*) and blotted dry with sterile filter paper. Explants were then transferred to selection medium (CSM: MP medium supplemented with appropriate antibiotics) under a 16-h photoperiod (3000 lx) at 25–28°C. Subculturing was performed every 14–16 days for 45–60 days until resistant calli emerged.

Recovery and Confirmation: Resistant calli were transferred to recovery medium (CHM: CSM with reduced timentin) and subjected to three rounds of subculture (14–16 days each) with gradually increasing timentin concentrations (50 mg·L^−1^ increments per passage) to eliminate residual bacteria. Putative transgenic calli were confirmed by qRT-PCR using gene-specific primers (Table S11).

The genetic transformation protocol for *Populus* spp. followed standardized methodologies [81]. Primer sequences used for qRT-PCR amplification were provided in Table S11.

### Oligonucleotide probe-mediated silencing assay and validation of *ApMYB2*, *ApWRKY26*, and *ApERF4* in *A. palmatum*

To conduct oligonucleotide probe-mediated gene silencing assays and validation for *ApMYB2*, *ApWRKY26*, and *ApERF4* in *A. palmatum*, the sequences of these target genes were first submitted to the Soligo online website to predict their respective oligonucleotide antisense strand-specific probes (AsODN); candidate probes for each gene were preliminarily screened, and their reverse-complemented sequences were used to obtain oligonucleotide sense strand-specific probes (sODN), with all antisense and sense strand probes synthesized by a biotechnology company. Using healthy *A. palmatum* seedlings with uniform growth as experimental materials, healthy new shoots with one bud and two leaves were collected, their stems were obliquely cut with a sterile knife, and the cut shoots were placed into solutions containing the probes, with the connection between the tube mouth and shoots sealed with parafilm; the treatment groups consisted of shoots inserted into solutions containing AsODN of *ApMYB2*, *ApWRKY26*, or *ApERF4*, while the control groups included shoots treated with corresponding sODN or sterile water, with five biological replicates for each group. For the specific process, preliminarily screened probes for each gene were diluted to 100 μM with ddH_2_O, with controls being ddH_2_O and sODN diluted to the same concentration; uniform-growing branches (with four to six leaves) were inserted into centrifuge tubes containing the solutions, sealed with parafilm, and placed in an artificial climate chamber. Subsequently, uniform branches were inserted into solutions containing the screened AsODN (for each gene) and control solutions, with leaves at the second leaf position were collected. Expression levels of *ApMYB2, ApWRKY26*, and *ApERF4* were analyzed via qRT-PCR in treatment and control groups to verify silencing success, along with detecting downstream gene expression and observing leaf color changes.

### DAP-seq sampling and data analysis

Genomic DNA was isolated using the Zoonbio DN12 kit (Zoonbio Biotech), followed by size-selected purification and end-repair pipeline (NEBNext Ultra II FS DNA Library Prep Kit) to generate high-quality DNA fragments for library construction. The pDAP-Halo-Kan-KPHS-ApMYB2 expression vector was constructed, followed by cell-free protein expression experiments in the wheat embryo system and WB assay (Promega, L326A). ApMYB2-expressed proteins were incubated with a chicken pawpaw genomic DNA library, and the eluted AffiniPure DNA was subjected to PCR plus different index junctions. The raw data were then processed using Bowtie 2 alignment software (version 2.4.5) against the *A. palmatum* reference genome (Langmead and Salzberg, 2012). MEME-suite analysis identified conserved DNA motifs within DAP-seq peaks, while HOMER (v4.11) annotated functional elements. Use deepTools2 to analyze the frequency of distribution of reads near the TSS, plot density profiles, and corresponding heatmaps. Count the distribution frequency of peaks near the TSS and visualize the distribution of peaks on chromosomes.

### Yeast one-hybrid screening for ApMYB2 regulatory factors

Interacting TFs governing ApMYB2 expression were identified through a custom Y1H cDNA library (Zoonbio Biotech, Nanjing). Prior to library screening, self-activation of the bait constructs (pAbAi-ApF3'H2 and pAbAi-ApMYB2) was evaluated to avoid false-positive results. The coding DNA sequences (CDS) of ApWRKY21, ApERF23, and ApMYB2 were ligated into the pGADT7 vector (AD-ApWRKY21, AD-ApERF23, AD-ApMYB2), while the promoter regions of ApF3'H2 and ApMYB2 were inserted into the pAbAi vector (pAbAi-ApMYB2). For self-activation assays, each bait vector was transformed into Y1HGold yeast cells, which were then plated on SD/-Ura medium supplemented with gradient concentrations of Aureobasidin A. Results showed that potential self-activation of pAbAi-ApF3'H2 was inhibited by 50 ng·ml^−1^ AbA, and self-activation of pAbAi-ApMYB2 was suppressed by 100 ng·ml^−1^ AbA; these concentrations were thus chosen for subsequent interaction validation.

For interaction assays, AD-ApWRKY21 + pAbAi-ApMYB2, AD-ApERF23 + pAbAi-ApMYB2, and AD-ApMYB2 + pAbAi-ApF3'H2 were co-transformed into Y1HGold cells, and protein-DNA interactions were evaluated on SD/−Leu selection plates supplemented with 50 ng·ml^−1^ AbA (for ApF3'H2-related assays) or 100 ng·ml^−1^ AbA (for ApMYB2-related assays). The pGADT7 empty vector co-transformed with pAbAi-ApF3'H2 or pAbAi-ApMYB2 served as the negative control, and no growth was observed in these controls under the above AbA concentrations, confirming that positive results in the test groups were not due to self-activation of the promoters. Putative positive clones were sequenced by Sangon Biotech Co., Ltd (Shanghai, China).

### Electromobility shift assay (EMSA)

Probe sequences used for EMSA are detailed in Table S11. Follow the instructions of LightShift® Chemiluminescent EMSA Kit for EMSA detection [82]. Binding reaction mixtures were prepared in accordance with manufacturer guidelines, incubated at room temperature for 15 min, followed by probe addition and further incubation at 15°C for 25 min. Reaction products were resolved via 6% native polyacrylamide gel electrophoresis in 0.5× Tris-borate-EDTA (TBE) buffer and subsequently electrophoretically transferred to positively charged nylon membranes. Biotinylated DNA probes were detected using streptavidin-horseradish peroxidase conjugates paired with a chemiluminescent substrate system.

### Microscale thermophoresis assays

Microscale thermophoresis (MST) was employed to quantify dose-dependent binding interactions between: (i) ApMYB2 and the *ApF3'H2* promoter, and (ii) ApERF4/ApWRKY26 proteins and the *ApMYB2* promoter, using established protocols [83]. Proteins were fluorescently labeled with the RED-NHS 2nd Generation Kit (Monolith) according to manufacturer protocols. Samples were loaded and analyzed on a Monolith NT.115 system (NanoTemper Technologies, Germany). Binding affinity constants (Kd) were derived through nonlinear regression analysis using Nano Temper Analysis software. Triplicate experiments (*n* = 3) were statistically analyzed.

### Dual-LUC

The full-length *ApMYB2*, *ApERF4*, and *ApWRKY26* were integrated into the vector *pGreenII 62-SK*, whereas the promoter sequences of *ApF3'H2* and *ApMYB2* were integrated into the vector *pGreenII 0800-LUC*. Recombinant plasmids were transformed into *A. tumefaciens* strain EHA105 and transiently expressed in *N. benthamiana* leaves. The experiment was carried out in three biological replicates.

## Supplementary Material

Web_Material_uhaf257
