# Supplementary figures and images for: The ApWRKY26/ApERF4-ApMYB2 module regulates anthocyanin accumulation for the seasonal leaf color transition in Acer palmatum
Source: Hortic Res. 2025 Sep 24;13(1):uhaf257. doi: 10.1093/hr/uhaf257 (PMC12860562; doi:10.1093/hr/uhaf257)

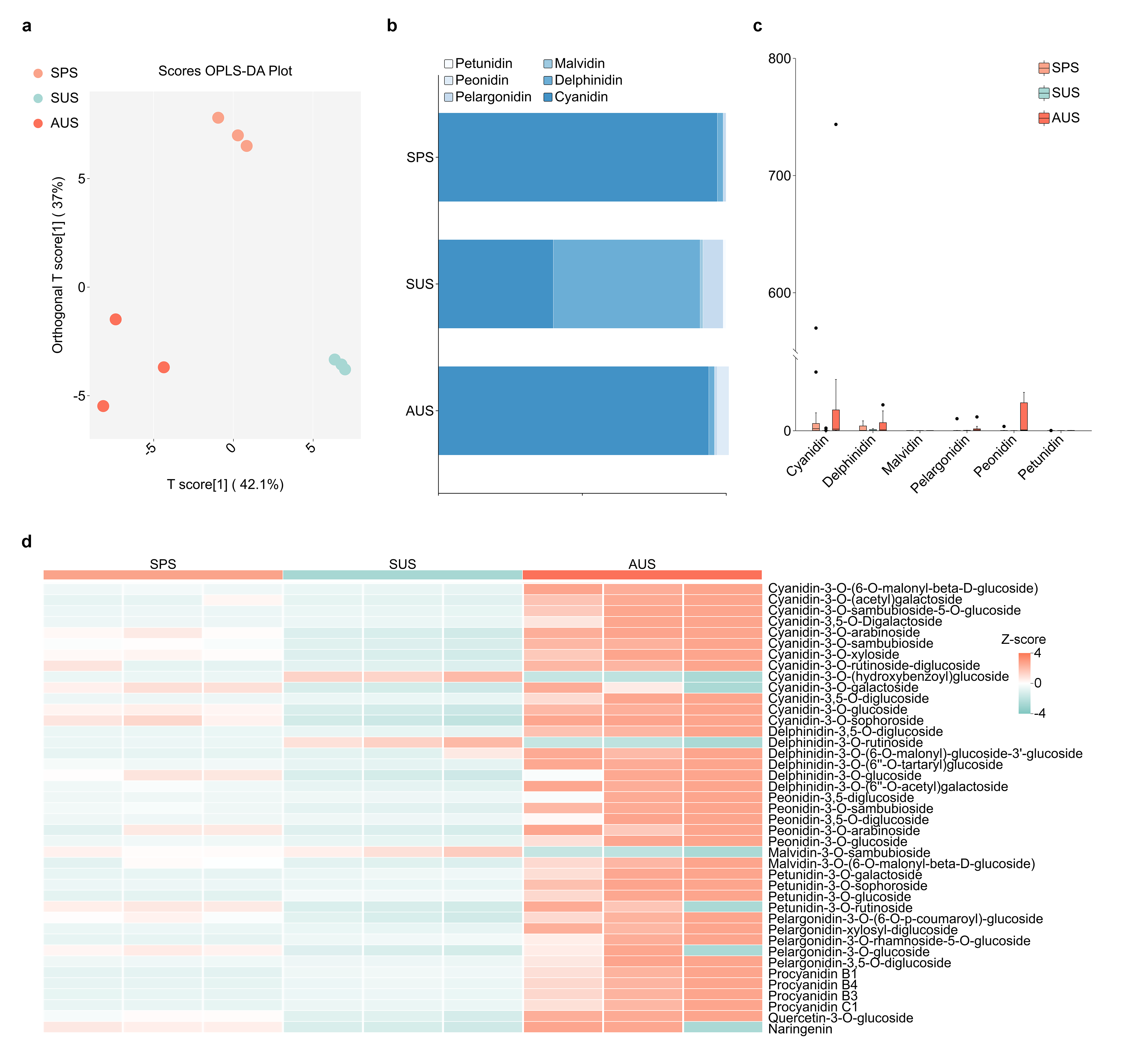

Supplement: Web_Material_uhaf257 [file web_material_uhaf257.zip › Extended Data Fig. 1 0715 lzw.tif]

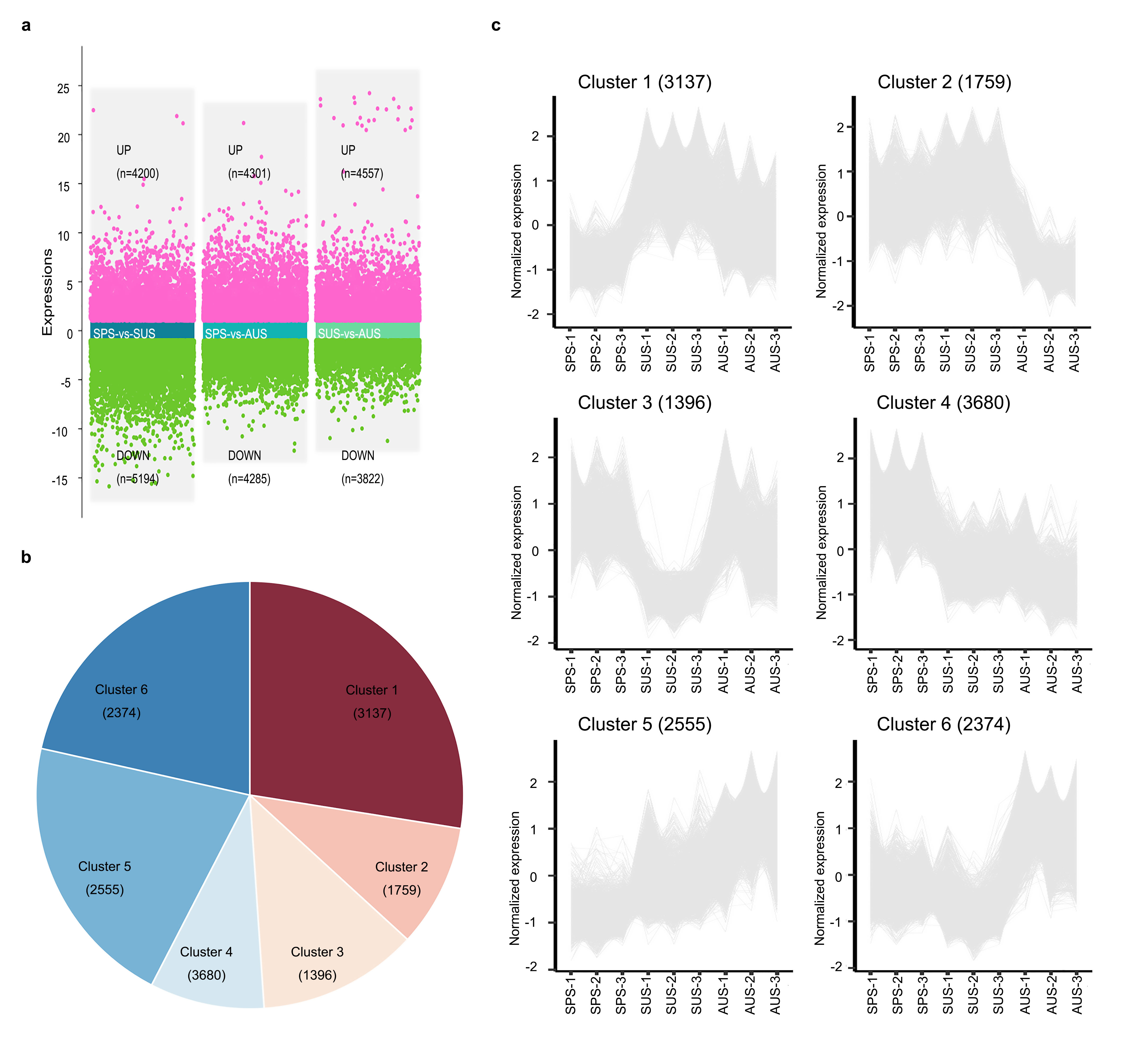

Supplement: Web_Material_uhaf257 [file web_material_uhaf257.zip › Extended Data Fig. 2.tif]

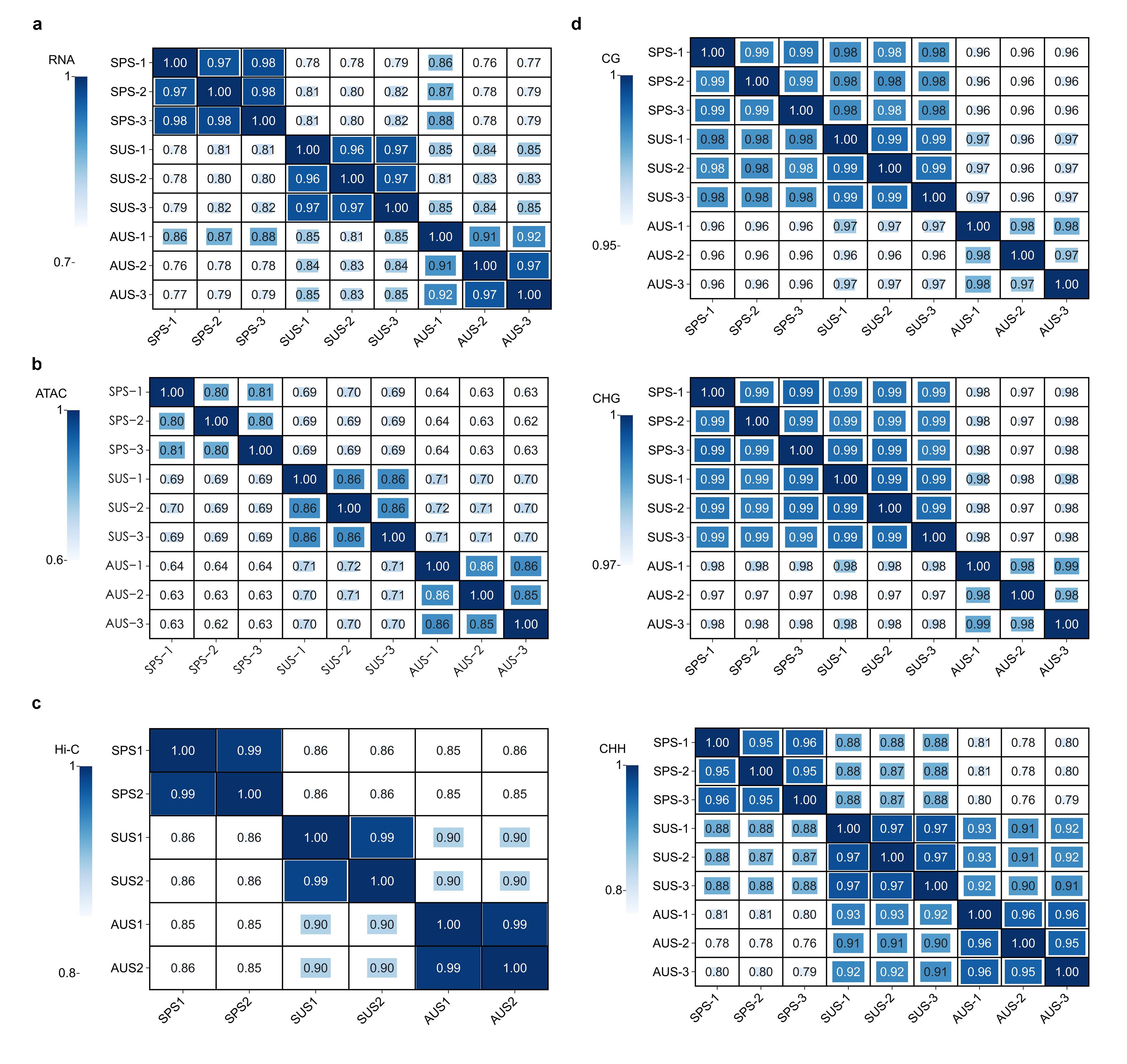

Supplement: Web_Material_uhaf257 [file web_material_uhaf257.zip › Extended Data Fig. 3 lzw.tif]

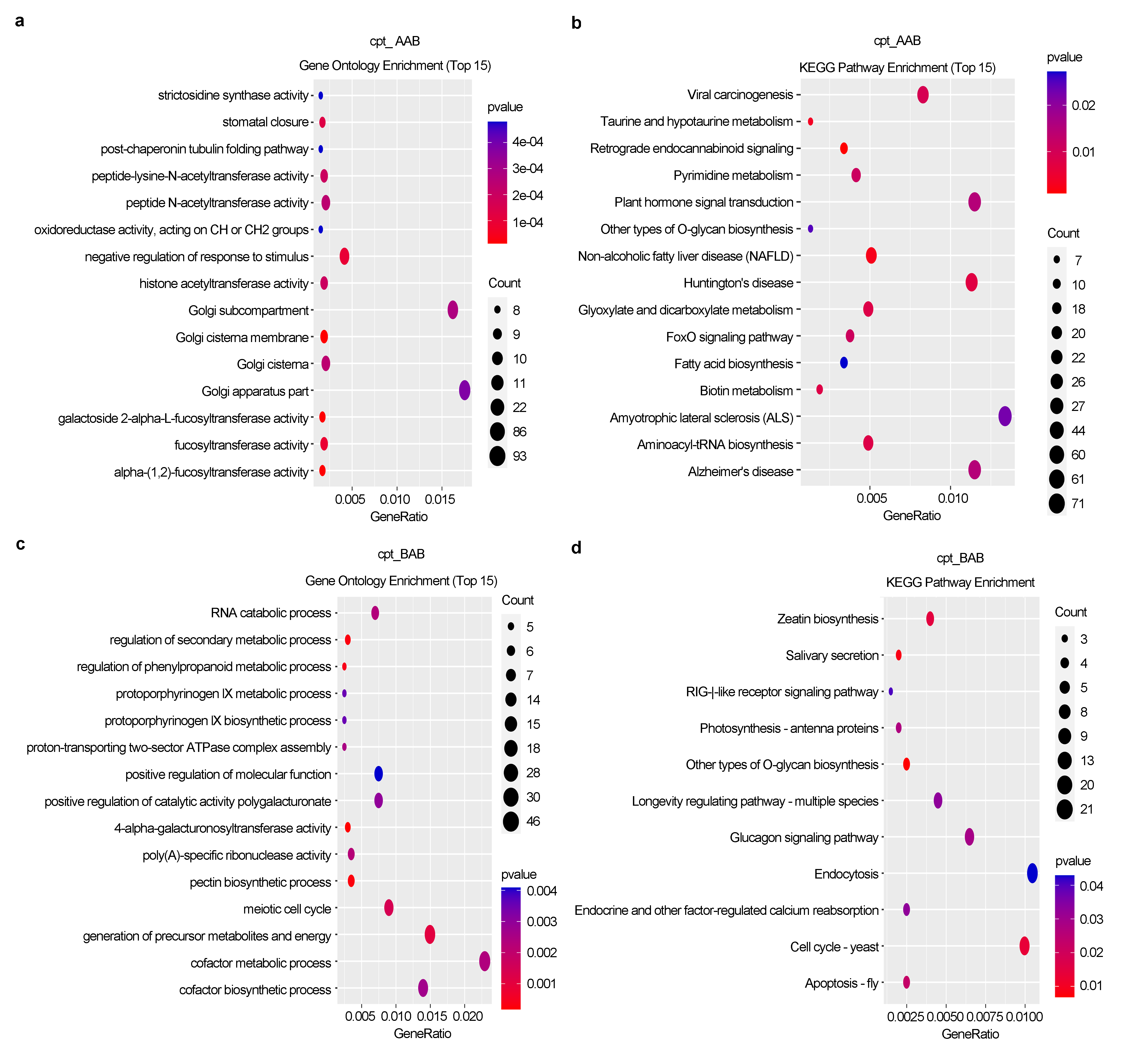

Supplement: Web_Material_uhaf257 [file web_material_uhaf257.zip › Extended Data Fig. 4 lzw.tif]

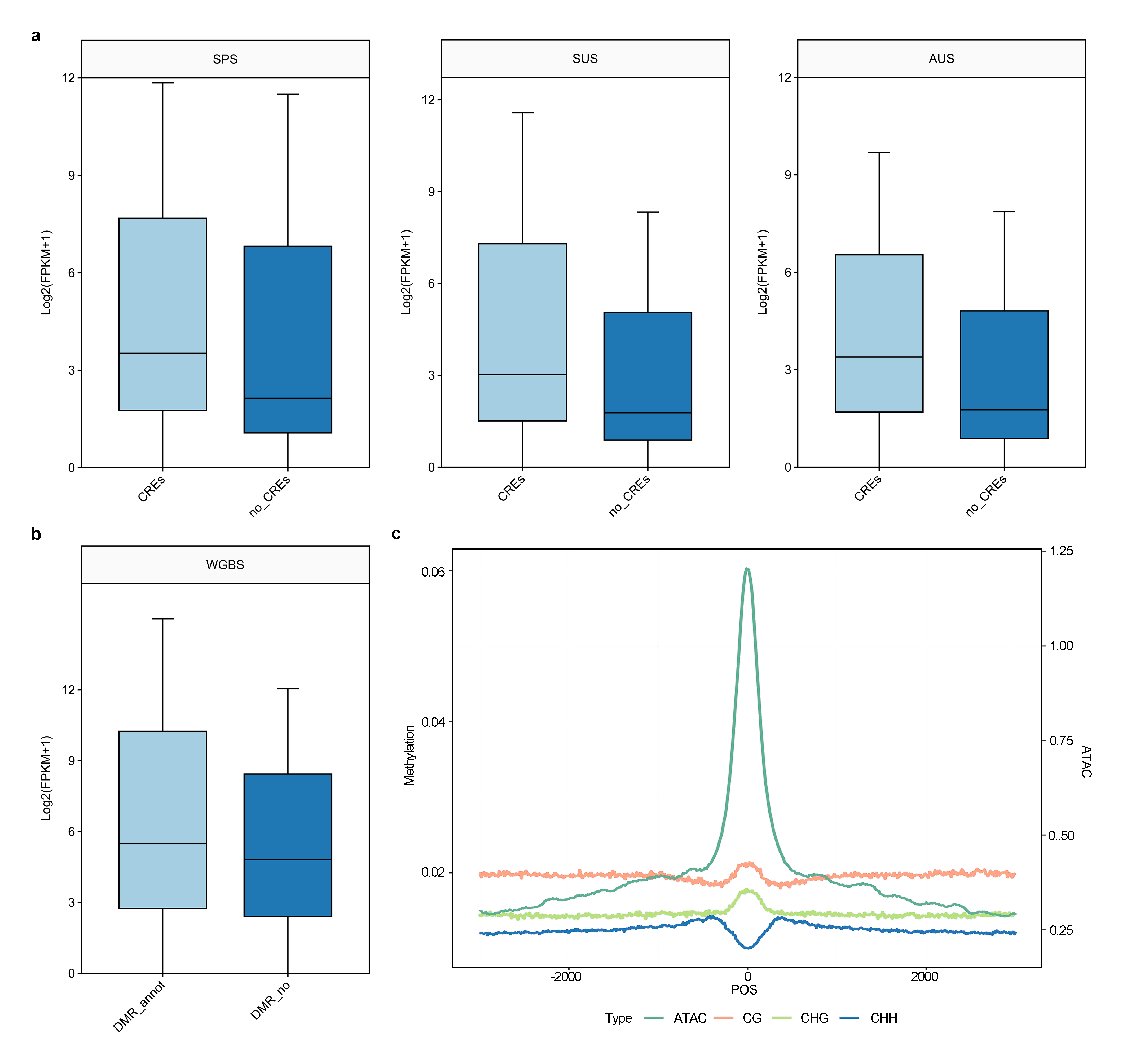

Supplement: Web_Material_uhaf257 [file web_material_uhaf257.zip › Extended Data Fig. 5 lzw.tif]

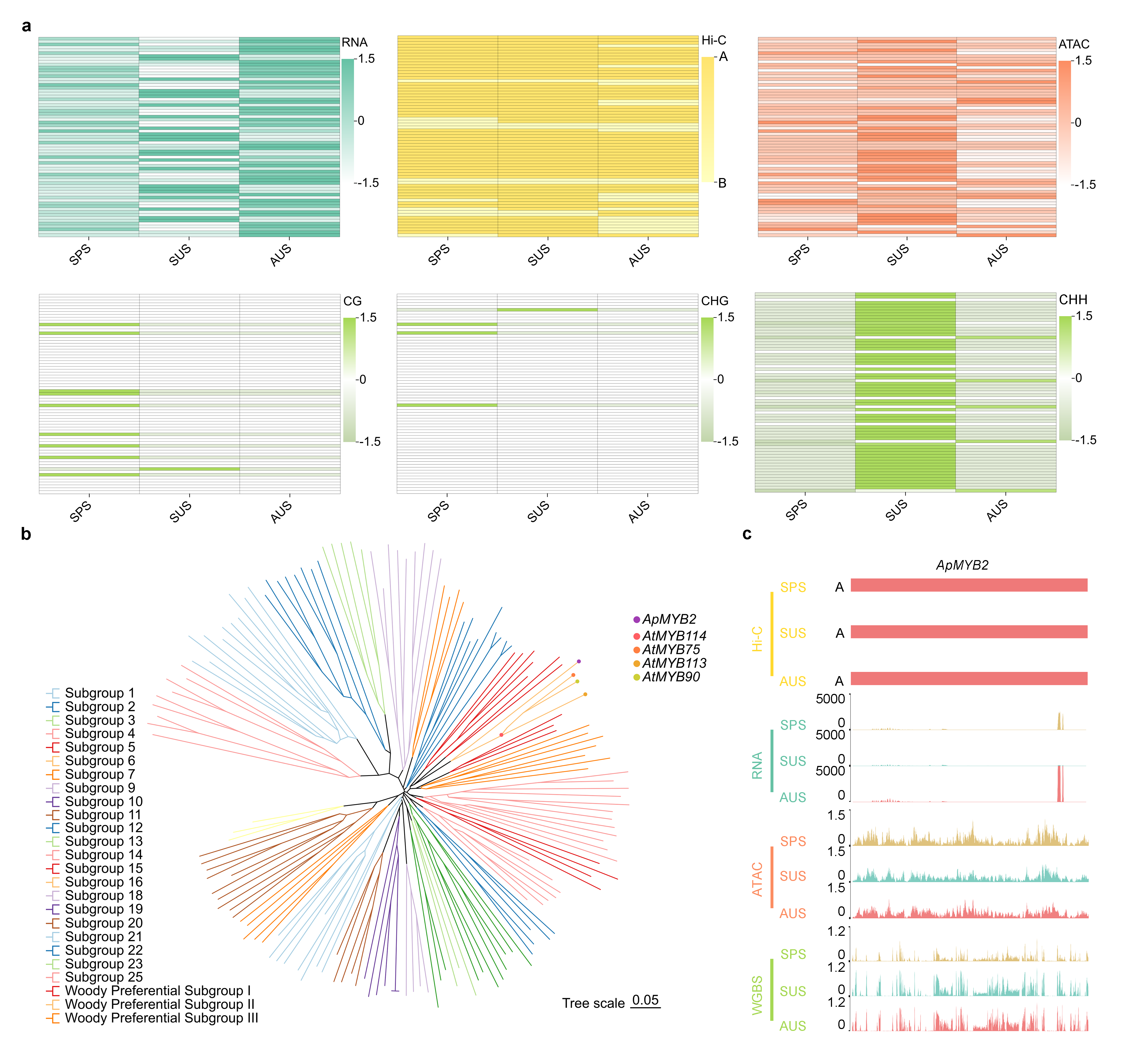

Supplement: Web_Material_uhaf257 [file web_material_uhaf257.zip › Extended Data Fig. 6 0715 lzw.tif]

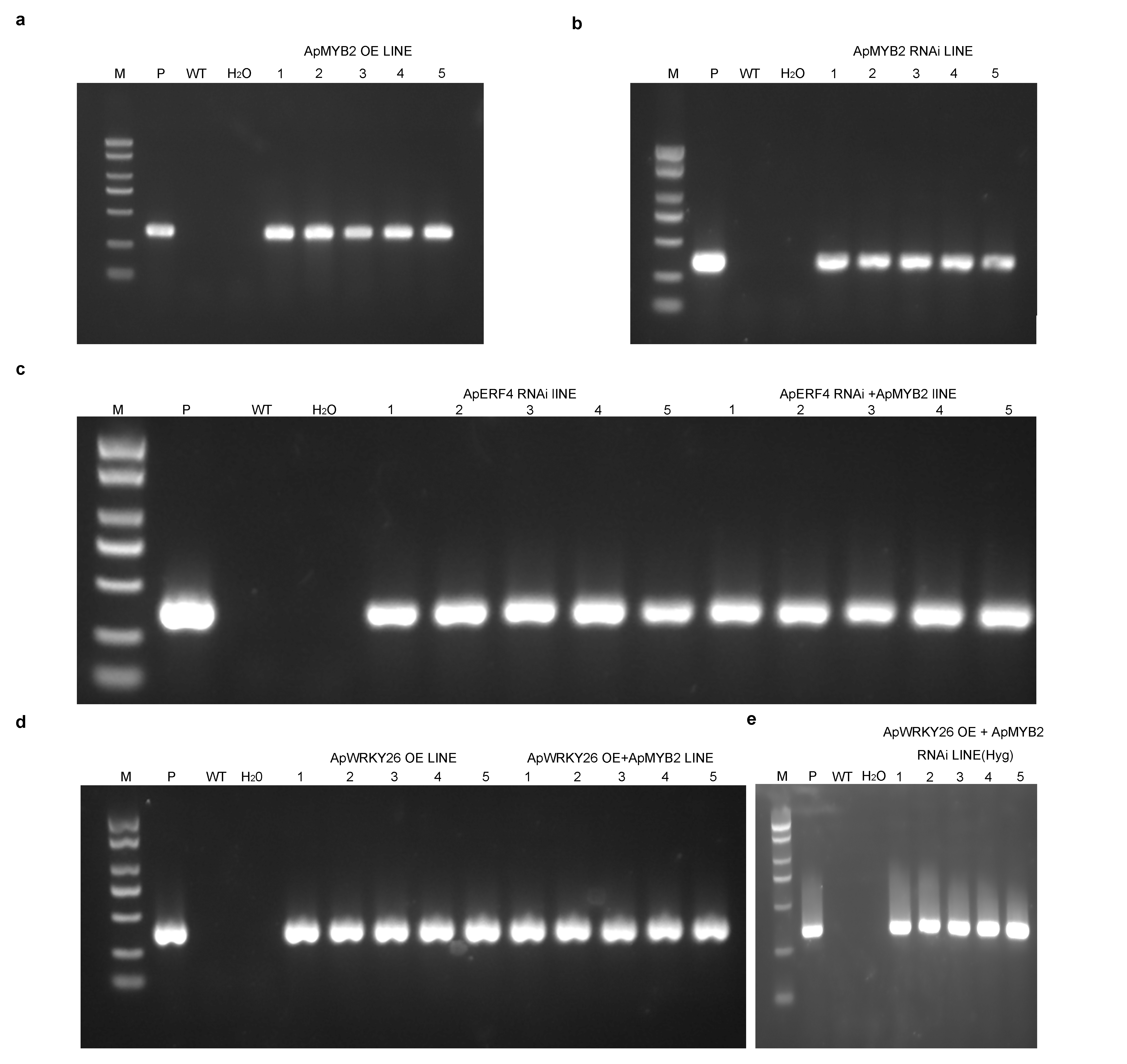

Supplement: Web_Material_uhaf257 [file web_material_uhaf257.zip › Extended Data Fig. 7 0715 lzw.tif]

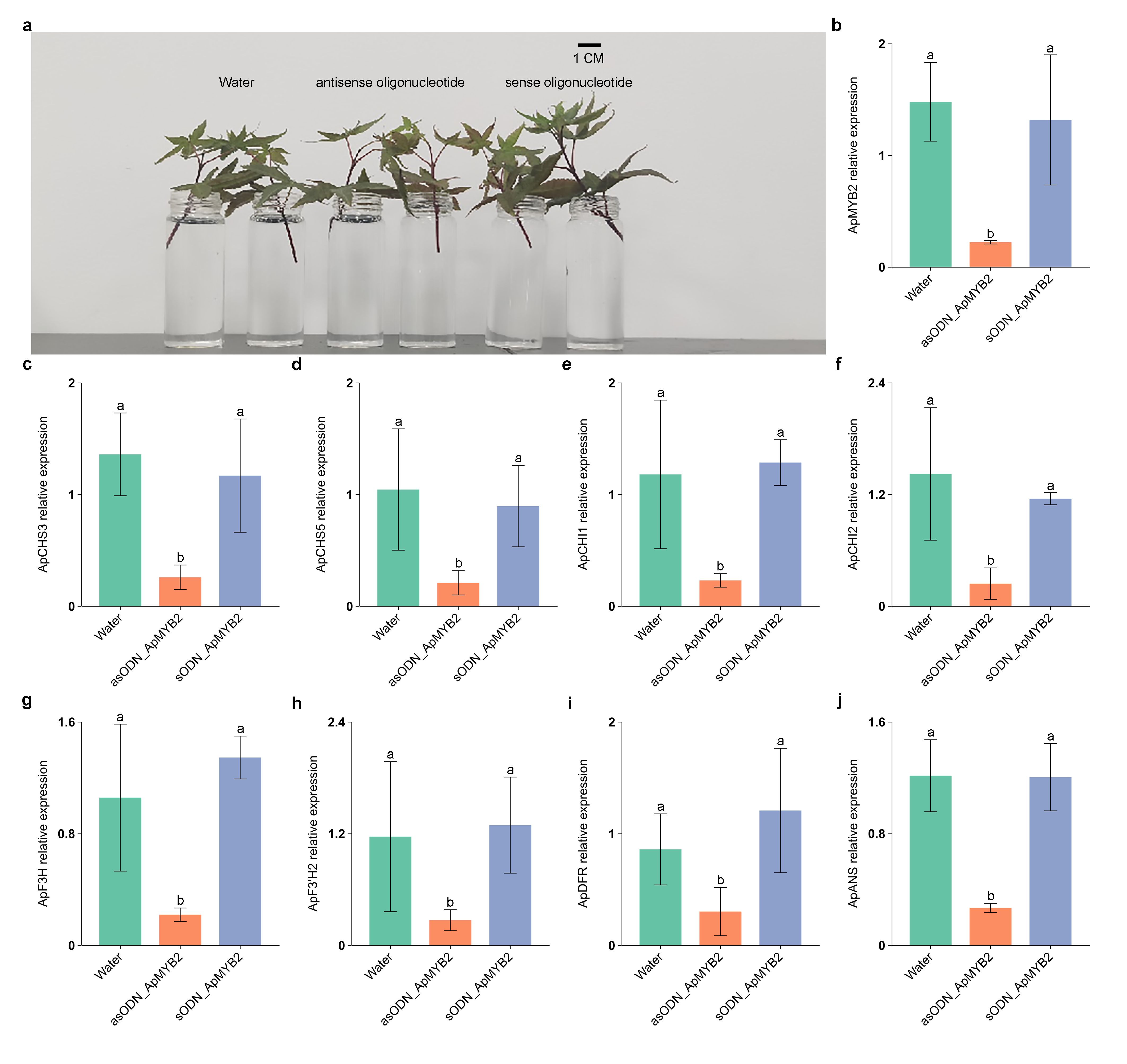

Supplement: Web_Material_uhaf257 [file web_material_uhaf257.zip › Extended Data Fig. 8 0715 lzw.tif]

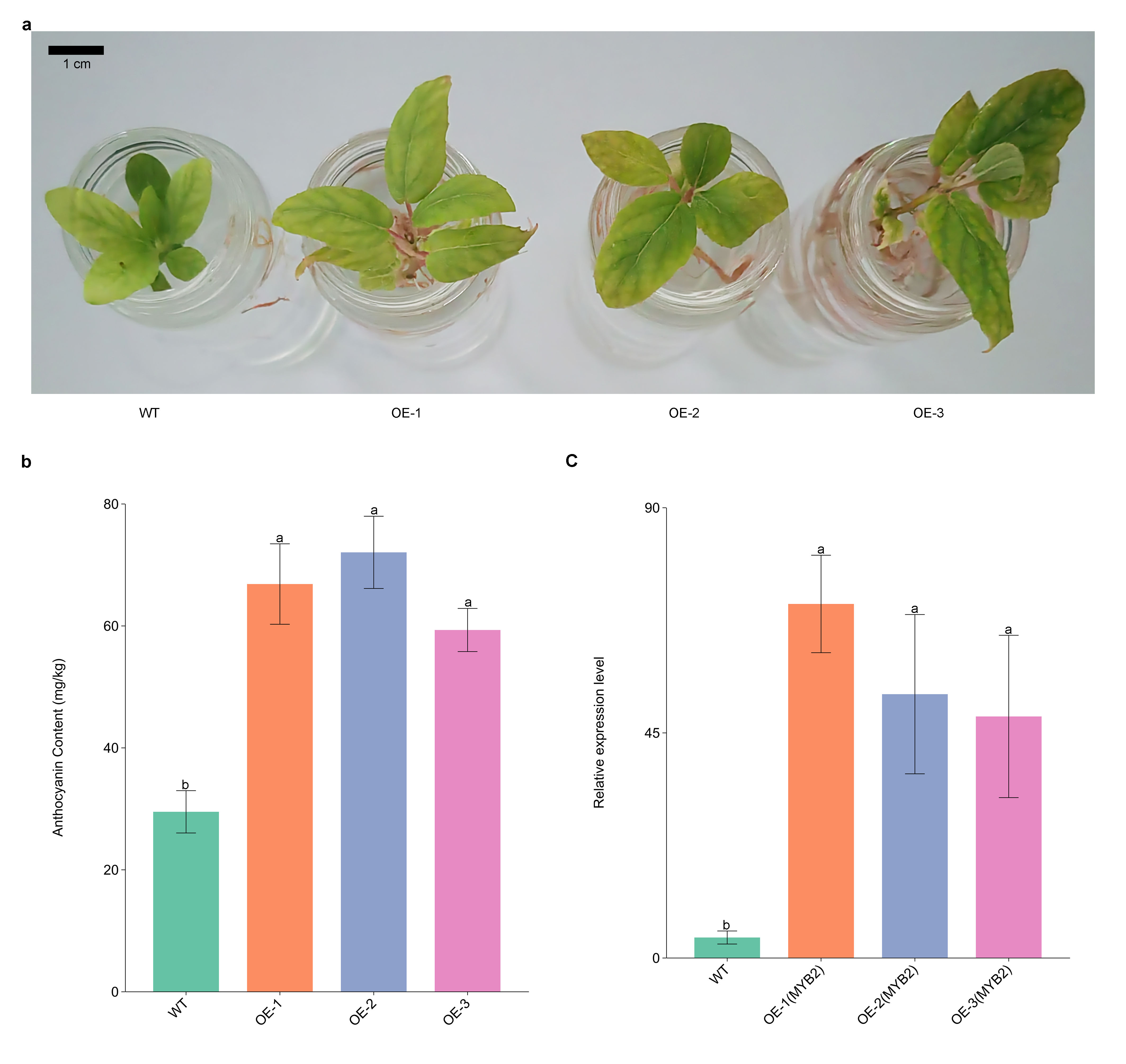

Supplement: Web_Material_uhaf257 [file web_material_uhaf257.zip › Extended Data Fig. 9 0715 lzw.tif]

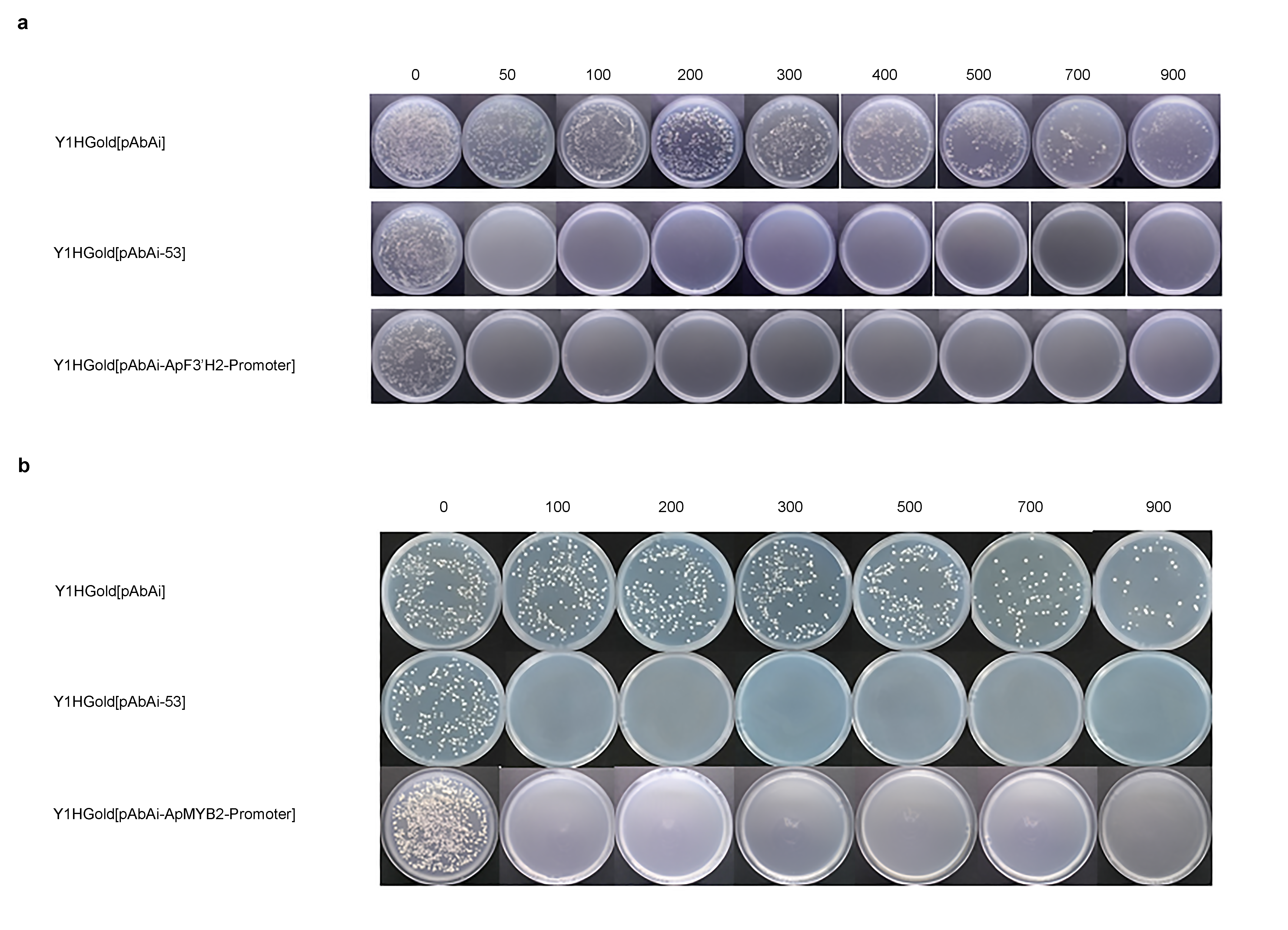

Supplement: Web_Material_uhaf257 [file web_material_uhaf257.zip › Extended Data Fig. 10 0715 lzw.tif]

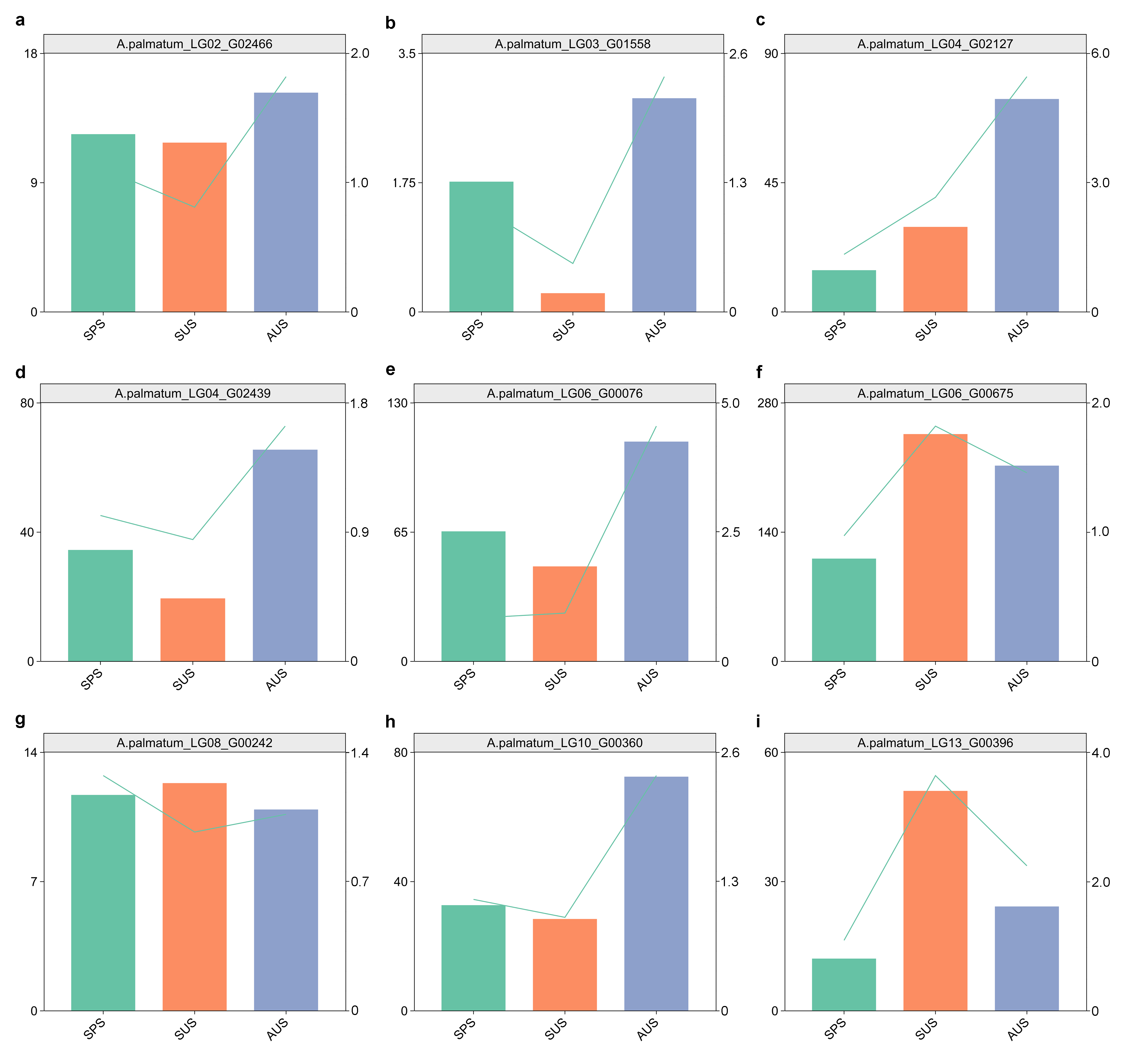

Supplement: Web_Material_uhaf257 [file web_material_uhaf257.zip › Extended Data Fig. 11 0715 lzw.tif]
